# Synthetic Dual Cysteine-ADP Ribosylated Peptides from the Androgen Receptor are Recognized by the DTX3L/PARP9 Complex

**DOI:** 10.1021/acschembio.3c00305

**Published:** 2023-11-08

**Authors:** Sven Wijngaarden, Chunsong Yang, Carlos Vela-Rodríguez, Lari Lehtiö, Herman S. Overkleeft, Bryce M. Paschal, Dmitri V. Filippov

**Affiliations:** † Leiden Institute of Chemistry, Leiden University, Einsteinweg 55, Leiden 2333 CC, The Netherlands; ‡ Department of Biochemistry and Molecular Genetics, University of Virginia School of Medicine, Charlottesville, Virginia 22908, United States; § Faculty of Biochemistry and Molecular Medicine and Biocenter Oulu, University of Oulu, Aapistie 7B, Oulu 90220, Finland

## Abstract

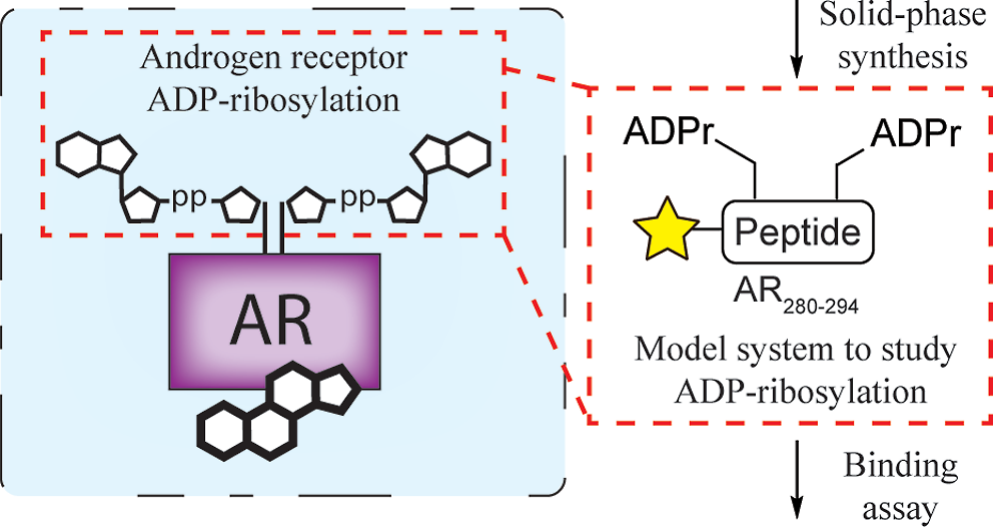

Androgen signaling in prostate cancer cells involves multisite cysteine ADP-ribosylation of the androgen receptor (AR) by PARP7. The AR modification is read by ADP-ribosyl binding macrodomains in PARP9, but the reason that multiple cysteines are modified is unknown. Here, we use synthetic peptides to show that dual ADP-ribosylation of closely spaced cysteines mediates recognition by the DTX3L/PARP9 complex. Mono and dual ADP-ribosylated cysteine peptides were prepared using a novel solid-phase synthetic strategy utilizing a key, Boc-protected, ribofuranosylcysteine building block. This synthetic strategy allowed us to synthesize fluorescently labeled peptides containing a dual ADP-ribosylation motif. It was found that the DTX3L/PARP9 complex recognizes the dual ADP-ribosylated AR peptide (*K*_d_ = 80.5 nM) with significantly higher affinity than peptides with a single ADP-ribose. Moreover, oligomerization of the DTX3L/PARP9 complex proved crucial for ADP-ribosyl-peptide interaction since a deletion mutant of the complex that prevents its oligomer formation dramatically reduced peptide binding. Our data show that features of the substrate modification and the reader contribute to the efficiency of the interaction and imply that multivalent interactions are important for AR-DTX3L/PARP9 assembly.

Modification of proteins with adenosine diphosphate ribose (ADPr) is facilitated by ADPr-transferase enzymes (e.g., ARTs) that transfer ADPr from NAD^+^ to nucleophilic acceptor amino acids Asp/Glu,^1,2^ Ser/Tyr,^3,4^ Arg,^5,6^ His,^7^ and Cys.^8−11^ This process is implicated in a number of normal and pathophysiological pathways. ADP-ribosylation of cysteine in prostate cancer cells, where modification occurs with mono-ADP-ribose (MAR), was recently shown to be a critical feature of a gene expression mechanism involving the androgen receptor (AR), a ligand-regulated transcription factor.^12^ Androgen binding to the ligand binding domain (LBD) of AR induces a conformational change of the unstructured N-terminal domain (NTD), enabling its interaction with the LBD. This process triggers AR transport into the nucleus where it is ADP-ribosylated on several cysteine residues by the writer enzyme PARP7.^12,13^ The agonist-bound, Cys-ADP-ribosylated AR is then recognized by the reader PARP9, which forms a heterodimer with the ubiquitin E3 ligase DTX3L via the Deltex binding domain (DeBD) on DTX3L.^12^ Thus, PARP7-mediated ADP-ribosylation of AR (writing) results in highly selective recruitment (reading) of DTX3L/PARP9 and modulation of AR-dependent gene expression.^12^ Point mutations in the PARP9 macrodomains (MDs) known to be critical for ADP-ribose binding abrogate DTX3L/PARP9 binding to AR.^12^

Our prior work showing that tandemly arranged MDs in PARP9 bind more efficiently to ADP-ribosylated AR than the individual macrodomains^12^ was the first clue that multivalent interactions might underpin DTX3L/PARP9 binding AR (Figure 1a). The observations that DTX3L/PARP9 assembles into an oligomer in vitro^14^ and that the native complex from prostate cancer cells has an apparent size >500 kDa^15^ both indicated that the complex that binds ADP-ribosylated AR contains multiple MDs; theoretically, these could engage multiple ADP-ribosyl Cys sites in AR. Such a mechanism would help explain the highly selective assembly of the AR-DTX3L/PARP9 complex, which is virtually undetectable prior to AR ADP-ribosylation by PARP7.^16^

**Figure 1:**
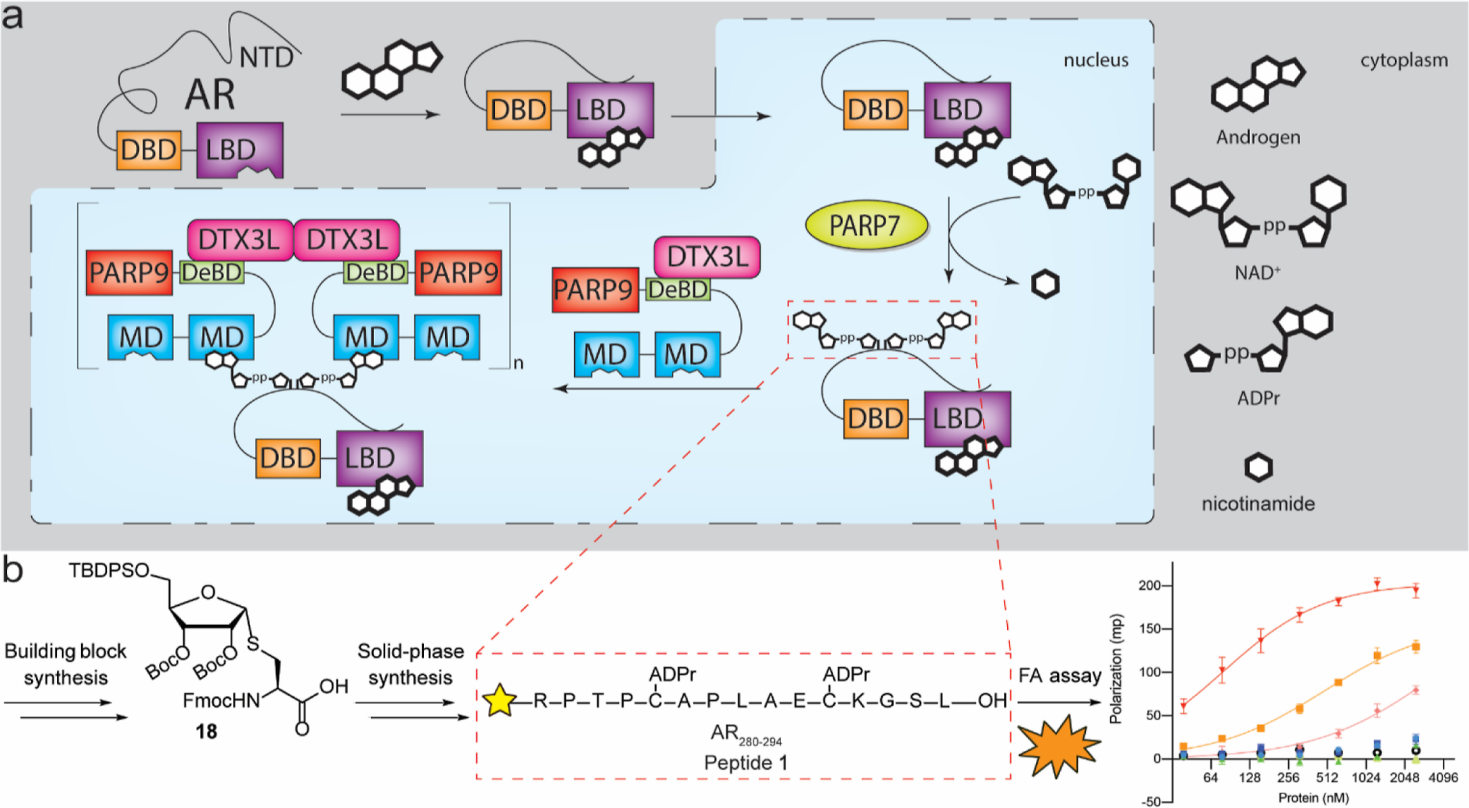
(a) Proposed mechanism of AR-DTX3L/PARP9 binding. Binding of androgen to the LBD of AR facilitates a conformational change, enabling the unstructured NTD to interact with the LBD. Hereafter, the complex is transported to the nucleus, where it gets ADP-ribosylated by PARP7. After ADP-ribosylation, the agonist-bound AR is recognized by the MDs on PARP9 which exists as an oligomeric heterodimer with DTX3L, leading to the modulation of AR-dependent gene expression. (b) Workflow for obtaining binding affinity of model peptide **1**. Solid-phase synthesis of dual-ADPr containing peptide **1** was based on building block **18**. Solid-phase peptide synthesis gave peptide **1** which could be tested for its affinity toward the oligomeric, heterodimerized reader complex DTX3L/PARP9.

Within the unstructured NTD of AR, four ADP-ribosylation sites (Cys 125 and Cys 131; Cys 284 and Cys 290) appear as pairs, each separated by six amino acids. Given that PARP9 contains two MDs, we postulated that the pairs of ADP-ribosyl Cys sites might be recognized by tandem MDs in PARP9. To address this question, we selected the Cys 284 and Cys 290 sites for focused analysis. Our preparation of bis-ADP-ribosylated AR_280–294_ (Figure 1b, peptide **1**) included developing an optimized synthesis scheme. To assess the importance of dual Cys-ADP-ribosylation in the context of binding the DTX3L/PARP9 oligomeric complex, a panel of mono-ADP-ribosylated peptides **2**–**5** were prepared. The binding affinities of Cys-ADPr peptides for the DTX3L/PARP9 reader complex were then measured by fluorescence polarization (FP). The results indicate that dual ADP-ribosylation of AR peptide is an essential determinant of ADP-ribose reading by the DTX3L/PARP9 complex.

We recently reported on the first chemical synthesis of MAR-peptides modified on cysteine.^17^ Target peptide **1** bearing two ADP-ribosyl-Cys residues, however, could not be prepared in this way (Figure S2), possibly due to the formation of multiple side products as a consequence of the increased complexity (two ADP-ribosyl-Cys units instead of one) that characterizes peptide **1** in comparison to the peptides we prepared previously. A major side product observed was the migration of a *para*-methoxybenzyl (PMB) protective group we had installed in our previous S-ribosyl cysteine building block **9** (see Scheme 1) to the cysteine thiol. A mechanistic review of this reaction led us to the proposed mechanism of migration (Scheme 1). TFA-induced protonation of the PMB ether leads to a cationic transition state, which can be intramolecularly intercepted by the sulfur via a 5- or 6-membered transition state. Hydrolysis of anomeric sulfonium species **11** then gives free ADPr and the *para*-methoxybenzylated cysteine.

**Scheme 1:**
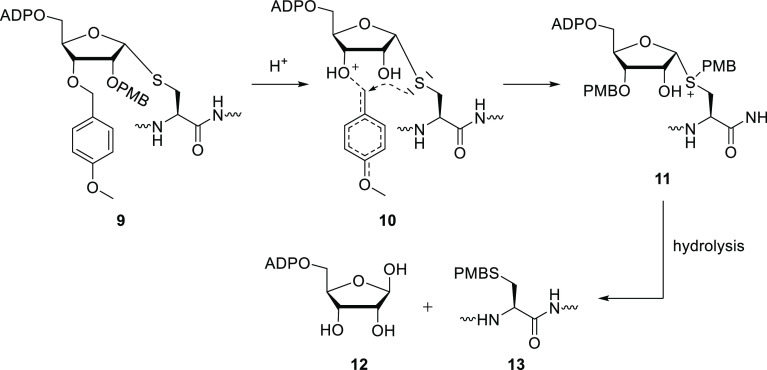
Proposed Mechanism of PMB Migration during Acidic Deprotection Protonation of the ether results in an attack of the thioether on the benzylic position, instigating PMB migration. Hydrolysis of the sulfonium salt **11** then gives the *para*-methoxybenzylated thiol of cysteine and free ADPr.

To circumvent PMB migration, building block **18** featuring Boc-protection of the 2′- and 3′-alcohols was envisioned (Scheme 2). The synthesis of **18** started with the fully α-selective glycosylation of the thiol in cysteine acceptor **14**, with trifluoroacetimidate ribosyl donor **15**. Addition of 10% (v/v) dioxane to the solvent (DCM) improved the solubility of the acceptor and sped up the reaction. Compound **16** was thus obtained with an 82% yield. PMB removal to give **17** in TFA/DCM proved abortive and again resulted in full migration of the PMB to the thiol to give **18** (Table 1). Oxidative deprotection of **16** with 2,3-dichloro-5,6-dicyano-1,4-benzoquinone (DDQ) in contrast, yielded **18** and the *para*-methoxybenzylidene derivative **17** (entry 1). Other acidic deprotection conditions, such as catalytic HCl in hexafluoroisopropanol (HFIP), gave PMB migration to side product **20** as well (entry 2).^18^ Deprotection of the PMB with 10% v/v TFA in DCM with the addition of ethane dithiol (EDT, **S2**) as a scavenger for the PMB cation gave an inseparable mixture of the desired product **17** with ribosylated EDT (**21**) (entry 3). Thioanisole (**S3**) and dimethyl sulfide (DMS, **S4**) as thioether scavengers gave inconsistent results (entries 4–5). Next, PMB deprotection in tetrahydrothiophene (THT, **S5**) as a solvent was examined (entry 6), and 30% TFA in THT gave the desired compound **17** in the highest yield (entries 7–8). Next, both alcohols in diol **17** were Boc-protected leading to the fully protected ribosylated cysteine, which after final deprotection of the allyl ester with Pd(PPh_3_)_4_ gave building block **18** ready for use in a solid-phase peptide synthesis.

**Scheme 2:**
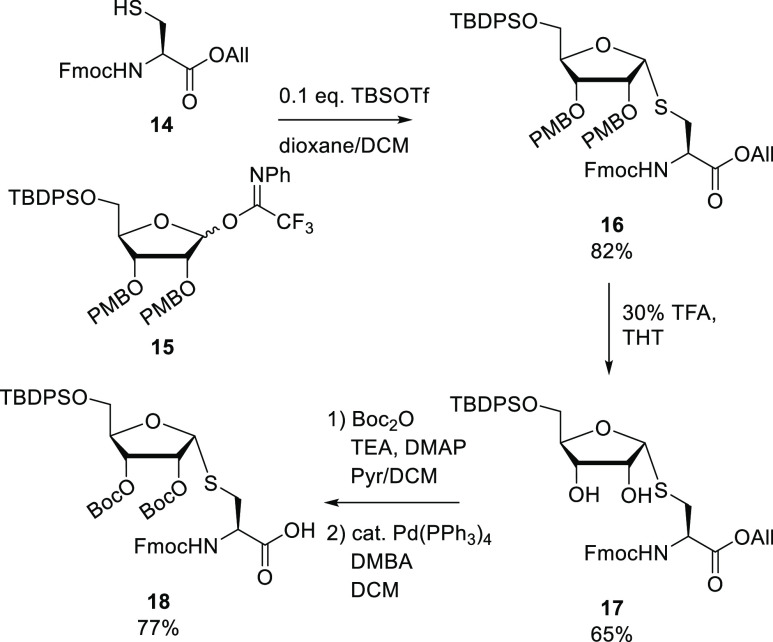
Synthesis of Suitably Protected Ribosylated Cysteine Building Block **18** Ready for Solid-Phase Synthesis

**Table 1:**
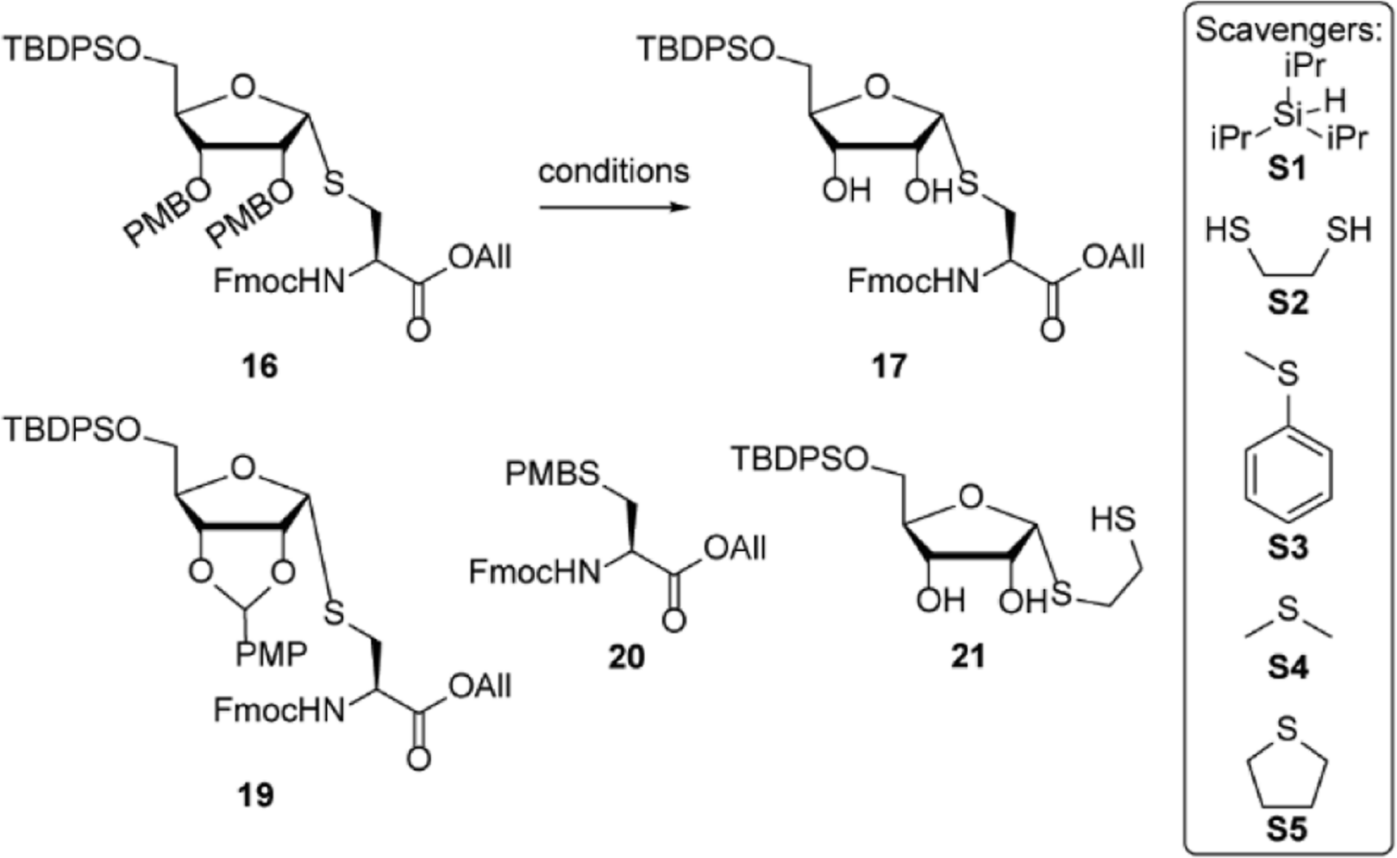
Optimization of PMB Deprotection toward Compound **17**^a^

#	reagent	solvent	scavenger^e^	yield	comments
**1**	3 equiv DDQ^b^	MeOH/DCM		N.A.	**19 + 20**
**2**	0.1 equiv HCl^c^	HFIP		N.A.	**20**
**3**	10% TFA^d^	DCM	**S1, S2**	54%	**+21**
**4**	10% TFA^d^	DCM	**S1, S3, S4**	61%	
**5**	10% TFA^d^	DCM	**S1, S3, S4**	27%	**0.5 mmol**
**6**	10% TFA^d^	THT	**S1**	31%	
**7**	30% TFA^d^	THT	**S1**	56%	
**8**	30% TFA^d^	THT	**S1**	65%	**1 mmol**

a Reactions were carried out at a concentration of 0.1 M and a scale of 0.1 mmol unless stated otherwise.

b Oxidative deprotection conditions (DDQ) resulted in only migration to PMB-cysteine **20** and *para*-methoxybenzylidene **19**.

c Catalytic HCl/HFIP resulted in complete migration to compound **20**.

d Acidic deprotection conditions using TFA and appropriate scavengers resulted in formation of **17**, as well as **20**.

e Cation scavengers were used in a 2.5% v/v ratio. Triisopropyl silane (TIS, **S1**), ethane dithiol (EDT, **S2**), thioanisole (**S3**), dimethyl sulfide (DMS, **S4**), and tetrahydrothiophene (THT, **S5**).

With building block **18** in hand, the synthesis of peptide **1** was undertaken (Scheme 3). Conventional Fmoc-SPPS chemistry was used for peptide elongation to obtain ribosylated and partially protected peptide **22** in the solid phase. Next, N-terminal (5)-carboxy fluorescein (CF) was installed using (7-Azabenzotriazol-1-yloxy)tripyrrolidinophosphonium hexafluorophosphate (PyAOP) activation.^19^ The self-acylation of CF on its phenolic alcohols was dealt with by treatment with 20% v/v piperidine/DMF to cleave the unwanted esters.^20^ The phenols of fluorescein were then protected with the trityl (Trt) group, enabling further on-resin modifications. Deprotection of the 5′-silyl ether of the ribose in **23** with tetra-butylammonium fluoride (TBAF) primed the construct for installation of the phosphate. Two key phosphorylation steps, utilizing phosphoramidite chemistry,^21^ were employed to yield the ADP-ribosylated peptide on-resin. First, the primary alcohol of ribose was phosphorylated using established phosphoramidite chemistry.^17,22^ Coupling of the alcohol with bis-Fm phosphoramidite **26** under activation of ethane 5-(ethylthio)-1*H*-tetrazole (ETT) yielded the phosphite triester P(III) intermediate. Notably, due to the acidic nature of the activator ETT, the Trt protecting groups installed previously on the phenols were partially deprotected, resulting in overphosphitylation. Usage of equimolar amounts of **26** and activator ETT prevented this undesired Trt cleavage and thus overphosphitylation. (1S)-(+)-(10-camphorsulfonyl)-oxaziridine)oxidation (CSO) of the P(III) intermediate to the P(V) phosphotriester and subsequent deprotection of the Fm groups using 1,8-Diazabicyclo(5.4.0)undec-7-ene (DBU) yielded phosphate monoester **24**. The pyrophosphate was then introduced by the phosphitylation of **24** with adenosine amidite **27**(23) followed by the oxidation of the P(V)–P(III) intermediate to the P(V)–P(V) pyrophosphate and 2-cyanoethyl (CNE) cleavage with DBU. The immobilized, partially protected ADPr-peptide was subjected to acidic global deprotection, giving the final Cys-ADPr peptide **1**. A critical feature of this route is the final acidic deprotection enabling a more conventional cleavage of the product off the solid support, while earlier routes toward ADPr-peptides made use of an alkaline final deprotection^24−26^ and mild acidic conditions.^17^ The relatively high stability of the thioglycoside linkage in Cys-ADPr permitted the use of a cleavage cocktail with 50% v/v TFA resulting in shorter deprotection times while keeping the quality of the peptide the same as with 10% v/v TFA. Final deprotection toward the ADPr containing construct **1** showed no protecting group migration to the thiol and drastically improved the quality of the synthesis (Figure S4 vs Figure S2). The tandem ADP-ribosylated peptide was isolated in a 4.4% yield. Mono-ADPr-peptide **3** and extended mono-ADPr-peptides **4–5** were prepared as described for **1** and isolated in good yields. Note that the usage of 50% TFA facilitated the cleavage of the relatively acid-stable Pbf protection from the arginine side chain. The other simpler control mono-ADPr-peptide **2** was readily accessed using our original method.^17^

**Scheme 3:**
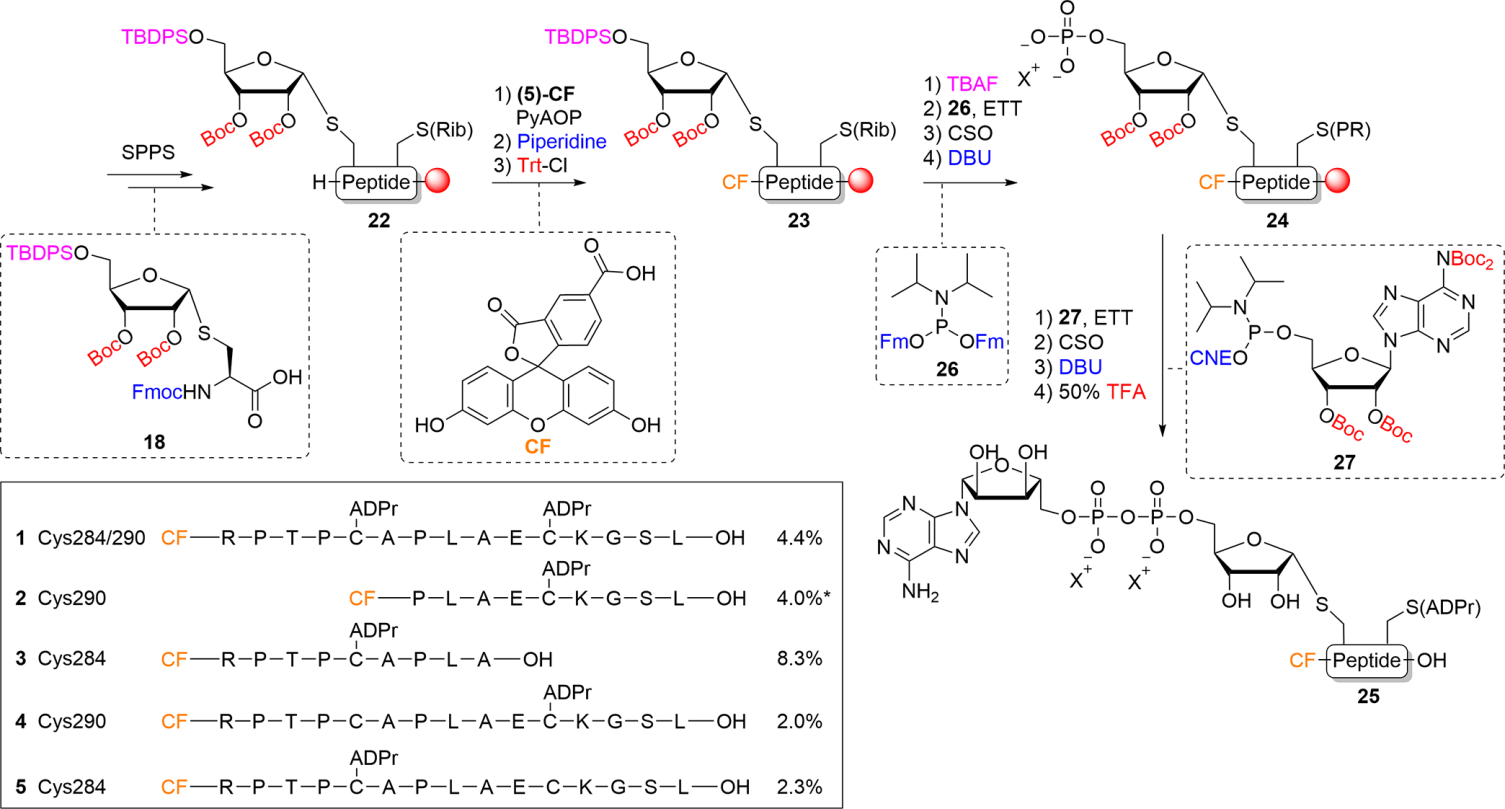
Improved Synthetic Strategy for the Synthesis of N-Terminally Modified, Tandem Cys-ADP-Ribosylated Peptides (Rib) corresponds to the appropriately protected ribosyl moiety and (PR) corresponds to the appropriately protected phosphoribosyl moiety. * Peptide **2** was synthesized via the procedure described by Voorneveld *et al.*(17)

To determine whether synthetic AR peptides containing one or two ADP-ribosylated Cys residues are recognized by the PARP9 MDs, we performed a series of binding reactions using fluorescent ADP-ribosylated peptides **1–5** and recombinant PARP9 and assayed the interactions by FP. We included recombinant DTX3L in the analysis, which heterodimerizes with PARP9 but also promotes formation of an oligomeric complex that based on gel filtration and light scattering contains at least four copies of the heterodimer.^14^ Because the PARP9 monomer encodes two MDs, the fully assembled DTX3L/PARP9 complex is predicted to contain eight MDs.

FP measurements revealed a low level of binding of PARP9 to the dual ADP-ribosylated AR peptide **1**, and binding was too weak to infer a *K*_d_ (Figure 2a). By contrast, DTX3L/PARP9, preassembled as a complex prior to peptide addition, displayed robust binding to peptide **1** (*K*_d_ = 80.5 nM). This differs from the near-background level of binding of DTX3L/PARP9 to AR peptides that contain a single ADP-ribose (peptides **2** and **3**). As expected, the control peptide **6** (Table S1) lacking ADP-ribose did not bind significantly to the DTX3L/PARP9 complex (Figure 2a). Thus, dual ADP-ribosylation of the AR peptide promotes binding to the DTX3L/PARP9 complex. To test if oligomerization of DTX3L/PARP9 affects its activity as an ADP-ribose reader, we took advantage of a DTX3L mutant that heterodimerizes with PARP9 but fails to form oligomers.^14^ DTX3LΔN/PARP9 displayed low binding to the dual ADP-ribosylated peptide **1** that was not saturable and likely reflects a weak association (*K*_d_ > 500 nM).

**Figure 2:**
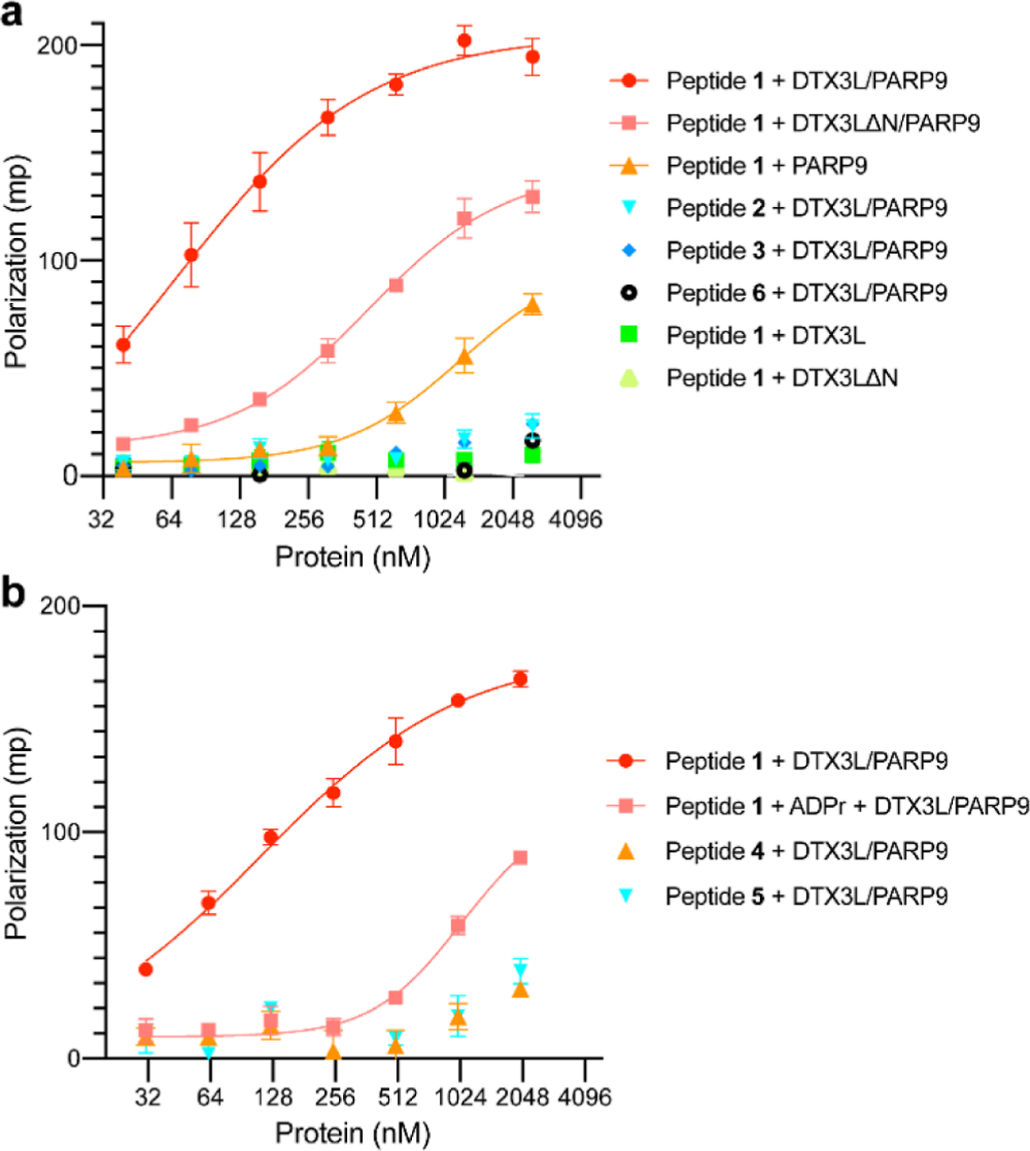
Interaction of DTX3L/PARP9 and ADP-ribosylated AR peptides measured by FP assays. (a) Oligomerization of DTX3L/PARP9 is critical for recognition of a dual ADP-ribosylated AR peptide. (b) DTX3L/PARP9 binding competes with ADP-ribose (50 μM) in solution. Assays were performed in triplicate in a 384-well plate (Corning 3575) containing 50 nM fluorescently labeled AR peptide and recombinant proteins [final concentration 2500 nM in (a) or 2000 nM in (b), and 2-fold serial dilution] in a binding buffer (20 mM Tris–HCl, pH 7.5, 50 mM NaCl, 0.1 mM EDTA, and 2 mM DTT). The reaction mixture was incubated at RT for 45 min, FP measured in a PHERAstar FSX Microplate Reader (BMG Labtech), and the binding curves were fitted for specific binding with GraphPad Prism v 9.3.1. The error bars represent the standard deviations. Dual-ADPr peptide **1**: CF-RPTPC(ADPr)APLAEC(ADPr)KGSL–OH; mono-ADPr peptides **2**: CF-PLAEC(ADPr)KGSL–OH; **3**: CF-RPTPC(ADPr)APLA–OH; **4**: CF-RPTCAPLAEC(ADPr)KGSL–OH; **5**: CF-RPTPC(ADPr)APLAECKGSL–OH. Control non-ADP-ribosylated peptide **6**: CF-RPTPCAPLAECKGSL–OH. Recombinant proteins were checked by SDS-PAGE as seen in Figure S10.

We then used the FP assay to confirm that the interaction between the dual ADPr peptide and DTX3L/PARP9 is competed by free ADP-ribose (Figure 2b). Addition of excess ADPr (50 μM) to the FP assay reduced the level of binding of DTX3L/PARP9 to dual ADP-ribosylated peptide **1** to ∼17% (Figure 2b). In addition, we used single ADP-ribosyl peptides (peptides **4** and **5**) that contain the same primary sequence as peptide **1**. This helped eliminate the concern that flanking sequences contributed to the superior binding observed with peptide **1** (Figure 2a,b). Overall, our ADP-ribosyl peptide binding results help extend our prior analysis showing that loss-of-function point mutations in the two PARP9 MDs (G112, 311E) eliminate binding to AR, as do mutations in the Cys ADP-ribosylation sites in AR that are modified by PARP7.^12^

Our data are consistent with a model of bivalent (and possibly multivalent) ADPr interaction with PARP9 that is dependent on DTX3L/PARP9 oligomeric assembly. We speculate that the spacing of the two Cys-ADPr moieties in the AR peptide may be too close to permit binding to the tandem MDs in a single PARP9 molecule. It seems plausible, however, that MDs juxtaposed in the DTX3L/PARP9 oligomeric complex may be sufficiently close to accommodate bivalent binding to the dual ADP-ribosylated-peptide. In any case, our reconstitution experiments suggest that multisite ADP-ribosylation is an important feature of AR binding to DTX3L/PARP9 and that bivalent interactions may be fundamental. The other pair of ADP-ribosyl-Cys sites in the N-terminal domain of AR (ADPr-C125, ADPr-C131), which were not included in this study, have the same spacing between the ADP-ribosylation sites as peptide **1** tested here by FP. These sites could potentially also be oriented in a manner that permits oligomeric DTX3L/PARP9 binding.

Because AR typically binds androgen response element DNA as a homodimer, other modes of DTX3L/PARP9 binding are conceivable, such as the oligomer using MDs to engage the NTDs of two adjacent AR molecules. These interactions might influence AR binding to DNA and also help position the DTX3L E3 ligase relative to the location of its substrates on chromatin. Depletion of DTX3L can have positive and negative effects on AR-dependent transcription,^12^ suggesting that reader function and E3 ligase activity are probably used in multiple ways. Finally, given that other transcription factors are ADP-ribosylated by PARP7, recognition of ADP-ribosyl by DTX3L/PARP9 could be important in other gene expression pathways. Whether other transcription factors undergo multisite ADP-ribosylation in a manner that promotes engagement with DTX3L/PARP9 remains to be explored.

In conclusion, our work reveals a new and effective strategy for the synthesis of fluorescently labeled Cys-ADPr-peptides, including the first preparation of a well-defined synthetic peptide containing a dual ADP-ribosylation motif. To facilitate synthesis of tandem ADPr-peptide **1**, in which a CF was introduced on the N-terminus, Boc-protected Fmoc-cysteine-ribofuranosyl building block **18** was designed and synthesized. Sequential desilylation, phosphorylation, and pyrophosphorylation then yielded peptide **1** without migration of the protecting group on ribose to the cysteinyl thiol. The availability of fluorescent peptides **1–5** derived from the AR and ADP-ribosylated on Cys 284, 290 allowed us to assess whether tandem ADP-ribosylation is an essential factor in the recognition of the AR by the DTX3L/PARP9 heterodimeric complex. The binding was determined via an FP assay. Binding of DTX3L/PARP9 to Peptide **1** gave a saturable binding in the nanomolar range (*K*_d_ = 80.5 nM), while insignificant binding was observed of peptides **2**–**5** with DTX3L/PARP9 indicating the importance of the dual ADPr motif. Binding of peptide **1** to the mutant DTX3LΔN/PARP9 heterodimer, which does not oligomerize, or PARP9 without DTX3L, resulted in binding that was not saturable. Together these observations lead us to posit that closely spaced ADP-ribosyl groups (e.g., Cys 284 and Cys 290) might not be recognized by the tandem MDs in a single PARP9 polypeptide but rather by the MDs’ positioned within the oligomer. It should also be noted that our analysis focused on a single pair of ADP-ribosylated Cys sites. Although the actual stoichiometry of ADP-ribosyl modified AR Cys sites has not been defined, it is conceivable that multiple sites are engaged by DTX3L/PARP9 at the same time. It is also possible that a subset of the ADP-ribosyl Cys sites has functions unrelated to DTX3L/PARP9 binding.

## Methods

### Solid-Phase Synthesis

#### Coupling CF

After peptide elongation, CF (2.5 equiv) and PyAOP (2.5 equiv) were suspended in DMF (0.25 M). NMP was added until the suspension was fully dissolved (0.1–0.15 M) and the mixture was added to the resin. DIPEA (5 equiv) was slowly added to the resin while shaking and the mixture was agitated overnight after it was washed with DMF and DCM. To cleave overacylated CF, the resin was treated with piperidine in DMF (20% *v*/*v*) and washed with DMF. The treatment was repeated after which the resin was washed with DMF and DCM. CF was protected with Trt by addition of Trt-Cl (11 equiv, 0.11 M) and DIPEA (11 equiv 0.11 M) in DCM to the resin, after which the mixture was shaken for 2 h, followed by washing with DCM.

#### On-Resin Deprotection and Phosphorylation

The resin was treated with TBAF in THF (1 M, 10 mL/g resin) for 30 min while shaking. The resin was washed with DMF and the treatment was repeated once, after which it was extensively washed with DMF and DCM yielding the desilylated ribosyl peptide intermediate. Hereafter, the resin was washed extensively with MeCN and flushed with N_2_ to remove all traces of water. A solution of **26** [(FmO)_2_PN(*i*Pr)_2_] (5 equiv, 0.25 M in MeCN) was added, followed by an ETT activator (5 equiv, 0.25 M in MeCN). The resin was agitated for 30 min, followed by washing with MeCN. A solution of CSO (8 mL/g resin, 0.5 M in MeCN) was added, and the mixture was shaken for 30 min, after which the resin was washed with MeCN and DCM. Hereafter, the Fm groups were deprotected by treatment of the resin with DBU in DMF (10% *v*/*v*) for 15 min followed by washing with DMF. The treatment was repeated after which the resin was washed with DMF, yielding the phosphoribosylated intermediate.

#### On-Resin Pyrophosphate Construction

The resin was extensively washed with MeCN and flushed with N_2_ to remove traces of water. The resin was treated with a solution of adenosine amidite **27** (3 equiv, 0.13 M in MeCN) and ETT (3 equiv, 0.25 M in MeCN) for 30 min while being agitated. The resin was thoroughly washed with MeCN before a CSO solution (0.5 M in MeCN) was added after which it was shaken for 30 min. The resin was washed with MeCN and DMF, and a solution of DBU in DMF (10% *v*/*v*) was added after which the resin was shaken for 10 min. The treatment was repeated once, after which the resin was washed extensively with DMF and DCM, yielding the ADPr-peptide on-resin.

#### Global Deprotection and Purification

The resin was globally deprotected and cleaved from the resin with cleavage cocktail (16 mL/g resin, 50% *v*/*v* TFA, 2.5% *v*/*v* TIS in DCM) for 1 h, after which it was filtered into cold Et_2_O (5 times the volume of cleavage cocktail). The resin was washed with a cleavage cocktail (2 mL/g resin), which was again filtered into the Et_2_O. The precipitated peptide was centrifuged for 5 min after which the supernatant was discarded. The resulting pellet was resuspended in Et_2_O and again centrifuged, followed by the removal of the supernatant. The crude peptide was dissolved in an NH_4_OAc solution of 1:1 Milli-Q:MeCN (100 mM), lyophilized, and purified by HPLC. Fractions containing the peptide were collected and lyophilized.

#### FP Assays

Recombinant PARP9, DTX3L, and DTX3LΔ*N* (N-terminal deletion mutant) were expressed in Sf21 insect cells and purified as previously described.^14^ Complex formation of DTX3L/PARP9 or DTX3LΔN/PARP9 was carried out in an equal-molar ratio by incubating the proteins at >1 mg mL^–1^ for each on ice for ∼4 h. Fluorescently labeled AR peptides (50 nM final concentration) **1**–**5** were mixed with various concentrations of the individual recombinant proteins or the protein complexes in a reaction buffer containing 20 mM Tris–HCl (pH 7.5), 50 mM NaCl, 0.1 mM EDTA, and 2 mM DTT, incubated at RT for 45 min and then measured in a PHERAstar FSX Microplate Reader (BMG Labtech). The assays were performed in triplicate. Competition assays were performed with 50 μM ADP-ribose. After correction for signals due to the fluorescent AR peptide alone, polarization values were plotted and analyzed with Prism (GraphPad) using nonlinear fit for specific binding with Hill slope.

## Supporting Information Available

Synthetic procedures and analysis data of key compounds and products, including NMR and HRMS analyses (PDF)
Raw data of the interaction of DTX3L/PARP9 and ADP-ribosylated AR peptides as measured by FP assays (XLSX)

## Abbreviations

ADPr: adenosine diphosphate ribose
AR: androgen receptor
ARTs: ADP-ribosyl transferase enzymes
CF: (5)-carboxy fluorescein
FP: fluorescence polarization
MAR: mono-ADP-ribose
MD: macrodomain
SPPS: solid-phase peptide synthesis
