# Supplementary material for: Synthetic Dual Cysteine-ADP Ribosylated Peptides from the Androgen Receptor are Recognized by the DTX3L/PARP9 Complex
Source: ACS Chem Biol. 2023 Nov 8;18(11):2377–84. doi: 10.1021/acschembio.3c00305 (PMC10660311; doi:10.1021/acschembio.3c00305)
Supplement: Supplementary file 1 — cb3c00305_si_001.pdf [file cb3c00305_si_001.pdf]

# Synthetic dual Cysteine-ADP ribosylated peptides from the Androgen Receptor are recognized by the DTX3L/PARP9 complex

Sven Wijngaarden,[a] Chunsong Yang,[b] Carlos Vela-Rodríguez,[c] Lari Lehtiö,[c] Herman S. Overkleeft,[a] Bryce M. Paschal,*[b] Dmitri V. Filippov*[a].

[a] Leiden Institute of Chemistry, Leiden University, Einsteinweg 55, 2333 CC Leiden, The Netherlands

[b] C. S. Yang, Prof. Dr. B. M. Paschal, Department of Biochemistry and Molecular Genetics, University of Virginia School of Medicine, Charlottesville, Virginia, 22908, USA

[c] C. Vela-Rodríguez, Prof. Dr. Lari Lehtiö, Faculty of Biochemistry and Molecular Medicine and Biocenter Oulu, University of Oulu, Aapistie 7B, 90220 Oulu, Finland

Email Bryce M. Paschal: paschal@virginia.edu

Email Dmitri V. Filippov: filippov@chem.leidenuniv.nl

# Contents

General synthetic procedure .......... 3
- Building block synthesis .......... 3
- Solid-phase synthesis .......... 7
  - General procedure peptide synthesis .......... 7
- List of synthesized peptides .......... 9

HP-LC/MS traces of crude MAR-peptides .......... 10
HP-LC/MS traces of purified MAR-peptides .......... 14
SDS Page gels: .......... 16
NMR spectra: .......... 16
References .......... 42

# General synthetic procedure

All reagents were of commercial grade and used as received unless stated otherwise. Solvents used in synthesis were dried and stored over 4 Å molecular sieves, except MeOH and MeCN which were stored over 3 Å molecular sieves. Column chromatography was performed on silica gel 60 Å (40-63 µm, Macherey-Nagel). TLC analysis was performed on Macherey-Nagel aluminium sheets (silica gel 60 $F_{254}$). Compounds were visualized on TLC by irradiation with UV (254 nm) and by spraying with either cerium molybdate spray (25 g/L $(NH4)_6Mo_7O_{24}$, 10 g/L $(NH4)_4Ce(SO_4)_4 \cdot H_2O$ in 10% $H_2SO_4$ water solution), $KMnO_4$ spray (20 g/L $KMnO_4$ and 10 g/L $K_2CO_3$ in water) or $H_2SO_4$ (20% $^{v}/_{v}$ in MeOH) followed by charring at c.a. 250˚C.

$^{1}$H and $^{13}$C NMR spectra were recorded on a Bruker AV-400, AV-500 or AV-600 NMR. Chemical shifts (δ) are given in ppm relative to tetramethylsilane as internal standard. Coupling constants (*J*) are given in Hz. For pyrophosphate containing peptides, a small amount of EDTA was added to the NMR sample to sharpen the peaks for $^{31}$P-NMR. All given $^{13}$C-APT spectra are proton decoupled and are presented with even signals (Cq. and $CH_2$) pointing upwards and odd signals (CH and $CH_3$) pointing downwards.

LC-MS analysis was performed on a Finnigan Surveyor HPLC system with a Nucleodur C18 Gravity 3 µm 50 x 4.60 mm column (detection at 200-600 nm) coupled to a Finnigan LCQ Advantage Max mass spectrometer with ESI or a Thermo Scientific Vanquish UHPLC coupled to a Thermo Scientific LCQ Fleet ion mass spectrometer with ESI. Buffers used were A= $H_2O$, B= MeCN and C= 1% TFA/$H_2O$. The methods used were 10→90% 13.5 min (0→0.5 min: 10% MeCN; 0.5→8.5 min: 10% to 90% MeCN; 8.5→11 min: 90% MeCN; 11→13.5 min: 10% MeCN) or 0→50% 13.5 min. HPLC purification was performed on a Gilson GX-281 preparative HPLC with a Gemini-NX 5u, C18, 110 Å, 250 x 10.0 mm column or on a Waters autopurifier HP-LC/MS system coupled to a Phenomenex Gemini 5µm 150x21.2 mm column. HRMS was recorded on Thermo Scientific Q Exactive HF Orbitrap mass spectrometer equipped with an electrospray ion source.

## Building block synthesis

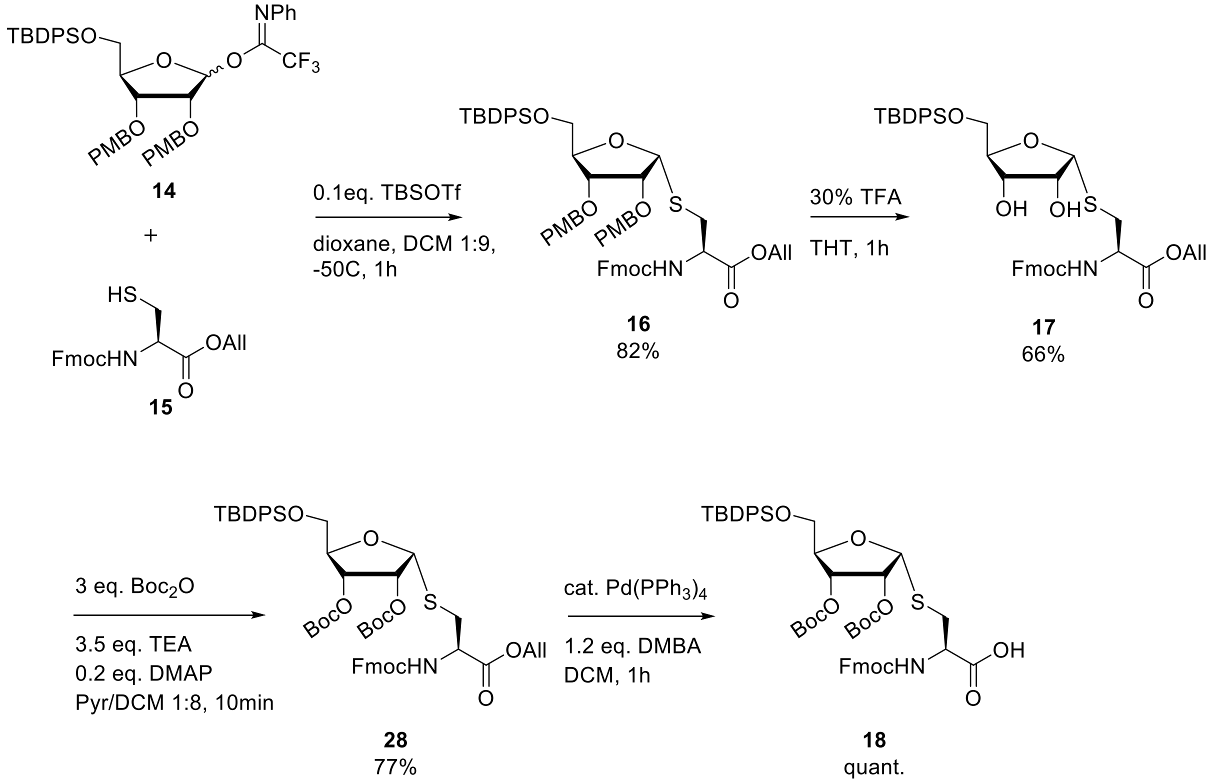


Scheme S1 synthetic scheme towards Fmoc-Cys-ribosyl building block **18**.

**1-*S*-2,3-bis-*O*-(4-methoxybenzyl)-5-*O*-((*tert*-butyl)-diphenylsilyl)-α-D-ribosyl)-*N*-fluorenylmethoxycarbonyl cysteine allyl ester (16)**

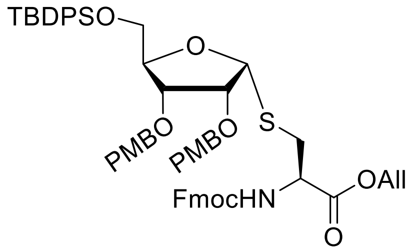


Acceptor **15** (1.71 mg, 4.45 mmol) and imidate donor **14** (3920 mg, 4.90 mmol, 1.1 eq.) were co-evaporated with toluene (3x) and dissolved in dioxane and DCM (1:9 $^{v}/_{v}$, 45 ml, 0.1M) and cooled to -50˚C. Activator TBSOTf (0.11 ml, 0.49 mmol, 0.11 eq.) was added and the mixture was stirred at -50˚C for 1 hour whereupon TLC analysis showed near full conversion of the starting material. The reaction was quenched with TEA and evaporated *in vacuo*. Purification by flash column chromatography (0% -> 0.5% -> 1.5% acetone/DCM) yielded the titled compound as a clear oil (3640 mg, 3.66 mmol, 82%). **Rf:** 0.47 (1.5% acetone/DCM). Spectra were in full accordance with literature experimental data.[1]

**1-*S*-5-*O*-((*tert*-butyl)-diphenylsilyl)-α-D-ribosyl)-*N*-fluorenylmethoxycarbonyl cysteine allyl ester (17)**

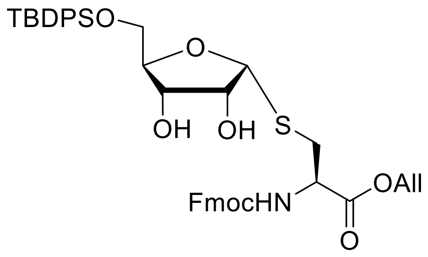


**16** (1020 mg, 1.02 mmol,) was dissolved in THT (7ml) after which a mixture of 50% TFA, 4% TIS in THT (14 ml) was added to a final concentration of 0.05M. The mixture was stirred for 1.5 hours, after which TLC analysis showed near full conversion of the starting material. The reaction was quenched with sat. aq. $NaHCO_3$ and extracted with DCM (2x). The combined organics were dried over $MgSO_4$, filtered, concentrated *in vacuo* and co-evaporated with toluene (3x). Purification by flash column chromatography (0% -> 2% -> 10% acetone/DCM) yielded the titled compound as a white foam (506 mg, 0.67 mmol, 66%). **Rf:** 0.40 (5% Acetone/DCM). **$^1$H NMR** (400 MHz, $CDCl_3$) δ 7.82 – 7.46 (m, 8H, Fmoc arom.+TBDPS arom.), 7.45 – 7.08 (m, 10H, Fmoc arom.+TBDPS arom.), 6.57 (d, *J* = 8.7 Hz, 1H, N*H*), 5.86 (ddt, *J* = 16.2, 10.7, 5.7 Hz, 1H, All), 5.41 (d, *J* = 5.4 Hz, 1H, *H*-1’), 5.33 – 5.12 (m, 2H. All), 4.73 (dt, *J* = 8.9, 4.7 Hz, 1H, $C_{\alpha}H$), 4.62 (d, *J* = 5.7 Hz, 2H, All), 4.47 – 4.31 (m, 2H, *H*-2’+ *H*-5’$_a$), 4.29 (t, *J* = 4.8 Hz, 1H, *H*-3’), 4.24 – 4.12 (m, 2H, *H*-4’+ C*H* Fmoc), 4.05 (t, *J* = 7.2 Hz, 1H, *H*-5’$_b$), 3.82 (d, *J* = 3.2 Hz, 2H, C*H₂* Fmoc), 3.73, 3.32 (dd, *J* = 14.5, 5.3 Hz, 1H, $C_{\beta}H_a$), 2.99 (dd, *J* = 14.5, 3.8 Hz, 1H, $C_{\beta}H_b$), 1.01 (s, 9H, TBDPS). **$^{13}$C NMR** (101 MHz, $CDCl_3$) δ 170.40 (C=O All), 156.39 (C=O Fmoc), 143.85, 143.70, 141.21 (*C*q. Arom.), 135.54, 135.50 (*C*H arom.), 132.93, 132.91 (*C*q. Arom.), 131.47 (All), 129.84, 129.78, 127.78, 127.74, 127.09, 127.03, 125.20, 125.08, 119.91 (*C*H arom.), 118.96 (All), 90.41 (*C*-1’), 83.88 (*C*-4’), 72.51 (*C*-2’), 71.46 (*C*-3’), 67.18 (*C*-5’), 66.32 (All), 63.66 ($CH_2$ Fmoc), 54.33 ($C_{\alpha}$H), 46.96 (*C*H Fmoc), 34.51 ($C_{\beta}H_2$), 26.82 (*t*Bu TBDPS), 19.23.5 (*C*q. TBDPS) **HRMS** [$C_{42}H_{47}NO_8SSi + H^+$] found: 754.2862, calculated: 754.2864.

**1-*S*-2,3-bis-*O*-(*tert*-butyloxycarbonyl)-5-*O*-((*tert*-butyl)-diphenylsilyl)-α-D-ribosyl)-*N*-fluorenylmethoxycarbonyl cysteine allyl ester (28)**

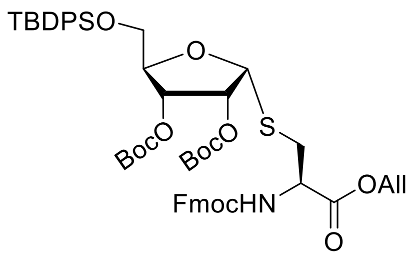


**17** (506 mg, 0.67 mmol) was dissolved in pyridine and DCM (1:8 % $^{v}/_{v}$, 6.7 ml, 0.1M). TEA (0.33 ml, 2.35 mmol, 3.5 eq.), $Boc_2O$ (0.47 ml, 2.02 mmol, 3.0 eq.) and DMAP (16 mg, 0.13 mmol, 0.2 eq.) were added and the solution was stirred for 15 minutes whereupon TLC analysis showed full conversion of the starting material. The reaction was quenched with 1M aq. HCl and extracted with DCM (2x), dried over $MgSO_4$, filtered, concentrated *in vacuo* and loaded on celite. Purification by flash column chromatography (10% -> 20% -> 30% EtOAc/pentane) yielded the titled compound as a white foam (495 mg, 0.52 mmol, 77%).

**Rf:** 0.65 (25% EtOAc/pentane). **$^1$H NMR** (400 MHz, $CDCl_3$) δ 7.77 – 7.48 (m, 8H, Fmoc arom.+TBDPS arom.), 7.42 – 7.18 (m, 10H, Fmoc arom.+TBDPS arom.), 6.36 (d, *J* = 8.7 Hz, 1H, NH), 5.90 (ddt, *J* = 16.3, 10.7, 5.8 Hz, 1H, All), 5.56 (d, *J* = 5.1 Hz, 1H, *H*-1'), 5.37 – 5.18 (m, 4H, *H*-2'+ *H*-3'+All), 4.75 (dt, *J* = 8.6, 4.2 Hz, 1H, $C_\alpha H$), 4.65 (d, *J* = 5.7 Hz, 2H, All), 4.45 – 4.30 (m, 3H, *H*-5'$_a$+C*H* Fmoc), 4.20 – 4.10 (m, 1H, *H*-5'$_b$), 4.07 (q, *J* = 7.3, 6.4 Hz, 1H, *H*-4'), 3.84 (d, *J* = 2.8 Hz, 2H, C*H*$_2$ Fmoc), 3.40 (dd, *J* = 14.5, 4.9 Hz, 1H, $C_\beta H_{2a}$), 3.01 (dd, *J* = 14.5, 3.8 Hz, 1H, $C_\beta H_{2b}$), 1.50 (s, 18H, Boc), 1.03 (s, 9H, TBDPS). **$^{13}$C NMR** (101 MHz, $CDCl_3$) δ 170.04 (C=O All), 156.05 (*C*=O Fmoc), 152.54, 152.18 (*C*=O Boc), 143.91, 143.74, 141.23, 141.19 (*C*q. arom.), 135.54 (*C*H arom.), 132.85, 132.80 (*C*q. arom.), 131.56 (All), 129.78, 129.74, 127.75, 127.64, 127.62, 127.06, 127.01, 125.21, 125.11, 119.88 (*C*H arom.), 118.90 (All), 88.02 (*C*-1'), 83.04, 82.82 (*C*q. Boc), 80.68 (*C*H Fmoc), 73.82, 72.87 (*C*-2'/3'), 67.03 (*C*-5'), 66.25 (All), 62.95 (*C*H$_2$ Fmoc), 54.18 ($C_\alpha$H), 47.00 (*C*-4'), 34.91 ($C_\beta H_2$), 27.69 (*t*Bu Boc), 26.79 (*t*Bu TBDPS), 19.22 (*C*q. TBDPS). **HRMS** [$C_{52}H_{63}NO_{12}SSi + H^+$] found: 954.3909, calculated: 954.3913.

### 1-*S*-2,3-bis-*O*-(*tert*-butyloxycarbonyl)-5-*O*-((*tert*-butyl)-diphenylsilyl)-α-D-ribosyl)-*N*-fluorenylmethoxycarbonyl cysteine (18)

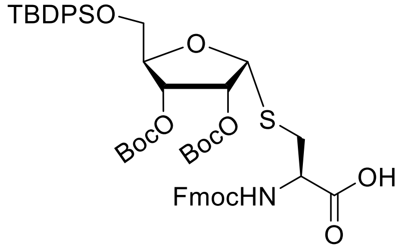


**28** (509 mg, 0.53 mmol) was dissolved in DCM. DMBA (100 mg, 0.64 mmol, 1.2 eq.) and a catalytic amount of $Pd(PPH_3)_4$ were added and the mixture was stirred for 1 hour before TLC analysis showed full conversion of the starting material. The reaction was diluted with 10% w/w aq. citric acid and extracted with DCM (2x). The combined organic layers were dried over $MgSO_4$, filtered and concentrated *in vacuo*. Purification by flash column chromatography (0% -> 0.5% ->10% MeOH/DCM) yielded the titled compound as a white foam (488 mg, 0.52 mmol, quant.). **Rf:** 0.22 (5% MeOH/DCM). **$^1$H NMR** (500 MHz, $CDCl_3$ + MeOD) δ 7.77 – 7.47 (m, 10H, arom. Fmoc+arom. TBDPS), 7.42 – 7.20 (m, 8H, arom. Fmoc+arom. TBDPS), 6.59 (d, *J* = 7.9 Hz, 0.3H, N*H*), 5.60 (d, *J* = 4.6 Hz, 1H, *H*-1'), 5.39 – 5.13 (m, 2H, *H*-2'/3'), 4.48 – 4.42 (m, 1H, $C_\alpha H$), 4.34 – 4.30 (m, 1H, C*H* Fmoc), 4.29 – 4.22 (m, 3H, *H*-5'$_a$/5'$_b$+$C_\alpha H$+C*H* Fmoc), 4.17 (dd, J = 10.4, 7.4 Hz, 1H, *H*-5'$_b$+*H*-4'), 4.03 (t, J = 7.2 Hz, 1H, *H*-4'), 3.80 (d, *J* = 3.0 Hz, 2H, C*H*$_2$ Fmoc), 3.39 – 3.32 (m, 1H, $C_\beta H_{2a}$), 3.07 (dd, *J* = 14.0, 3.9 Hz, 1H, $C_\beta H_{2b}$), 1.51 – 1.41 (m, 18H, *t*Bu Boc), 1.02 (s, 9H, *t*Bu TBPDS).**$^{13}$C NMR** (126 MHz, $CDCl_3$ + MeOD) δ 175.04 (*C*=O COOH), 156.56 (*C*=O Fmoc), 152.43, 152.19 (*C*=O Boc), 143.69, 143.66, 141.05 (*C*q. arom.), 135.37 (*C*H arom.), 132.66, 132.61 (*C*q. arom.), 132.32, 132.30, 131.88, 131.79, 129.65, 129.61, 128.65, 128.55, 127.58, 127.50, 126.89, 124.96, 119.70 (*C*H arom.), 87.83 (*C*-1'), 82.97, 82.86 (*C*q. Boc), 80.15 (*C*H Fmoc), 73.96, 72.96 (*C*-2'/3'), 66.80 (*C*-5'), 62.71 (*C*H$_2$ Fmoc), 54.74 ($C_\alpha$H), 46.83 (*C*-4'), 34.73 ($C_\beta H_2$), 27.41, 27.39 (*t*Bu Boc), 26.52 (*t*Bu TBPDS), 18.99 (*C*q. TBDPS). **HRMS** [$C_{49}H_{59}NO_{12}SSi + H^+$] found: 914.3596, calculated: 914.3600.

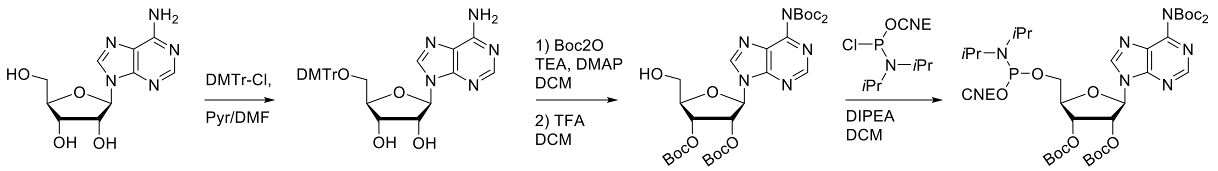


adenosine | 29 37% | 30 68% | 27 97%

Scheme S2 *Synthesis of* adenosine amidite *building block* **27.**

**5-*O*-(4,4'-Dimethoxytrityl) adenosine (29)**

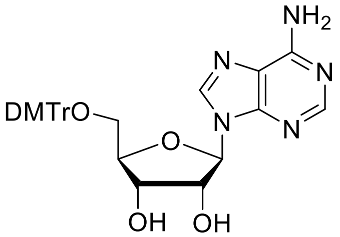

Adenosine (16.3 g, 40 mmol) was dissolved in Pyr and DMF (1:1, 80ml, 0.5M) and cooled to 0˚C. DMTr-Cl (16.3 g, 48 mmol. 1.2 eq.) was added in portions over 8 hours. Upon completion, the solution was crashed out as a yellow solid in cold $H_2O$ (800ml), filtered, washed with cold $H_2O$ and dried on air. The crude mixture was recrystallized from hot xylene (1000ml), giving a white precipitate after cooling to 0˚C which was filtered and washed with cold Et2O. The filtrate was collected and dried on a high vacuum at 60˚C, giving titled compound as a white solid (8.46 g, 14.6 mmol, 37%). Spectra were in full accordance with literature experimental data.[2]

**2,3-bis-*O*-(*tert*-butyloxycarbonyl)-*N*$^6$-di(*tert*-butyloxycarbonyl) adenosine (30)**

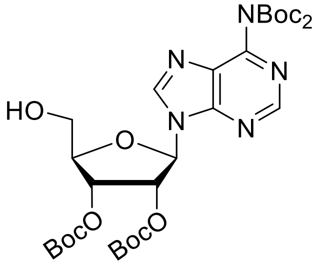

**29** (5.82 g, 10 mmol) was dissolved in DCM (100 ml, 0.1M). TEA (11.4 ml, 82 mmol, 8 eq.), $Boc_2O$ (14.2 ml, 60 mmol, 6 eq.) and DMAP (0.25 g, 2.0 mmol, 0.2 eq.) were added and the solution was allowed to stir over night. After TLC analysis showed full conversion of the starting material, the reaction was diluted with DCM, washed with sat. aq. $NaHCO_3$, dried over $MgSO_4$, filtered and concentrated *in vacuo.* The crude mixture was dissolved in DCM (146 ml, 0.07M). TIS (3.14 ml, 15 mmol, 1.5 eq.) and TFA (3.15 ml, 41 mmol, 4 eq.) were added and the mixture was stirred for 30 minutes. Upon completion as determined by TLC analysis, the reaction was quenched by addition of sat. aq. $NaHCO_3$ and extracted with DCM. The combined organic layers were washed with brine, dried over $MgSO_4$, filtered and concentrated *in vacuo*. Purification by flash column chromatography (20% -> 30% -> 50% EtOAc/pentane) yielded the titled compound as a white foam (4.63 g, 6.9 mmol, 68%). **Rf:** 0.49 (40% EtOAc/pentane). Spectra were in full accordance with literature experimental data.[3]

**5'-*O*-(2-cyanoethyl-*N*-*N*-diisopropylphosphoramidite)-2,3-bis-*O*-(*tert*-butyloxycarbonyl)-*N*$^6$-di(*tert*-butyloxycarbonyl) adenosine (27)**

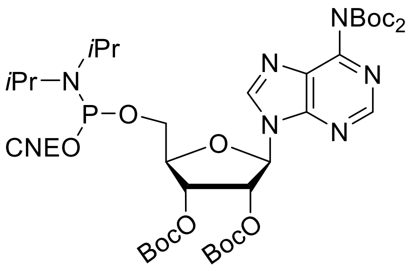

**29** (4.63 g, 6.94 mmol) was dissolved in DCM (70 ml, 0.1M). DIPEA (2.42 ml, 13.9 mmol, 2 eq.) and 2-cyanoethyl-*N*-*N*-diisopropylchlorophosphoramidite (1.70 ml, 7.63 mmol, 1.1 eq.) were added and the solution was stirred for 30 minutes until TLC analysis showed full conversion of the starting material. The reaction was quenched with MeOH and concentrated *in vacuo*. Purification by flash column chromatography (20% -> 30% EtOAc/pentane + 0.5% TEA) yielded the titled compound as a

white foam (5.84 g, 6.7 mmol, 97%). **Rf:** 0.49 (30% EtOAc/pentane). Spectra were in full accordance with literature experimental data.[3]

## Solid-phase synthesis

### General procedure peptide synthesis

The amino acids (obtained from Novabiochem and Sigma Aldrich) applied in the synthesis were: Fmoc-Ala-OH, Fmoc-Arg(Pbf)-OH, Fmoc-Cys(Trt)-OH, Fmoc-Glu(O*t*Bu)-OH, Fmoc-Glu(*O*-2-Ph*i*Pr)-OH, Fmoc-Gly-OH, Fmoc-Leu-OH, Fmoc-Lys(Boc)-OH, Fmoc-Lys(Mmt)-OH, Fmoc-Pro-OH, Fmoc-Ser(O*t*Bu)-OH, Fmoc-Ser(Trt)-OH, Fmoc-Thr(O*t*Bu)-OH, Fmoc-Thr(Trt)-OH, Fmoc-Cys(Trt)-OH. 5-carboxyfluorescein was bought from Bio-Connect B.V. TentaGel® S AC was bought from Rapp-Polymere GmbH and loaded by hand with the appropriate Fmoc-amino acid. ADPr peptides were synthesized with automated solid phase peptide synthesis on an CEM Liberty Blue Automated Microwave Peptide Synthesizer.
Automated synthesis of ADPr-peptides was performed on 25 or 50 µmol scale. The first amino acid was manually loaded on the resin using 2 eq. Fmoc-AA-OH, 2 eq. DIC and cat. DMAP while shaking over night. Resin was first swollen for 5 minutes in DMF prior to amino acid coupling. Activation was achieved using DIC/Oxyma. Standard coupling was achieved using 5 eq. Fmoc-amino acid as a 0.2 M DMF solution, 5 eq. DIC as a 0.5 M of DIC/DMF solution and 5 eq. Oxyma as a 1M solution in DMF which was buffered by DIPEA (0.1M) at room temperature for 60 minutes. Non-commercially available ribosylated Fmoc-amino acids were coupled using 3 eq. amino acid as a 0.1M solution in DMF, 3 eq. DIC as a 0.5M of DIC/DMF solution and 3 eq. Oxyma as a 1 M Oxyma/DMF solution which was buffered by DIPEA (0.1M) at room temperature for 120 minutes. Standard Fmoc deprotection was achieved by 20% $^{v}/_{v}$ piperidine/DMF at RT for 10 minutes (2 cycles). Synthesis quality could be monitored by UV absorption of dibenzofulvene released during Fmoc deprotection.

**CF-Arg-Pro-Thr-Pro-Cys(5-O-adenosine-diphosphate-α-D-ribosyl)-Ala-Pro-Leu-Ala-Glu-Cys(5-O-adenosine-diphosphate-α-D-ribosyl)-Lys-Gly-Ser-Leu-OH (1)**

The general procedures were followed as described to 50 µmol Tentagel® S AC resin unless stated otherwise. The amino acids used were Fmoc-Arg(Pbf)-OH, Fmoc-Pro-OH, Fmoc-Thr(O*t*Bu)-OH, Fmoc-Leu-OH, Fmoc-Ala-OH, Fmoc-Glu(O*t*Bu)-OH, Fmoc-Lys(Boc)-OH, Fmoc-Gly-OH, Fmoc-Ser(O*t*Bu)-OH and ribosyl building block **16**. For Phosphorylation, a solution of $(FmO)_2PN(iPr)_2$ (10 eq., 0.5M in MeCN) and ETT activator (10 eq., 0.5M in MeCN) was used. For pyrophosphorylation, a solution of adenosine amidite **16** (6 eq., 0.25 M in MeCN) and ETT (6 eq., 0.5 M in MeCN) was used. After HPLC purification, compound **1** was obtained as an orange powder (6.72 mg, 2.25 µmol, 4.5%).**$^1$H NMR** (600 MHz, $D_2O$ + $CD_3CN$) δ 8.62 (s, 2H, H-2), 8.47 – 8.44 (m, 1H, CF), 8.38 – 8.32 (m, 2H, H-8), 8.19 (t, *J* = 6.6 Hz, 1H, CF), 7.55 – 7.49 (m, 1H, CF), 7.22 (m, 2H, CF), 7.04 – 6.93 (m, 2H, CF), 6.92 – 6.83 (m, 2H, CF), 6.20 (m, 2H, H-1'), 5.72 – 5.63 (m, 0.2H, H-1''), 5.58 (d, *J* = 5.2 Hz, 1.3H, H-1''), 5.18 – 5.13 (m, 0.1H, H-1''), 5.11 (d, *J* = 5.4 Hz, 0.4H, H-1''). **$^{31}$P NMR** δ -10.33, -10.38, -10.43, -10.48, -10.56, -10.59, -10.62, -10.69. **LC-MS** (10% -> 50% MeCN) Rt = 4.78 min. **HRMS** [$C_{116}H_{163}N_{29}O_{52}P_4S_2 + 3H^+$] found: 995.3212, calculated: 995.3214.

**CF-Pro-Leu-Ala-Glu-Cys(5-O-adenosine-diphosphate-α-D-ribosyl)-Lys-Gly-Ser-Leu-OH (2)**

The general procedures described in Voorneveld et al.[1] were applied to 25 µmol Tentagel® S AC resin unless stated otherwise. The amino acids used were Fmoc-Pro-OH, Fmoc-Leu-OH, Fmoc-Ala-OH, Fmoc-Glu(O*t*Bu)-OH, Fmoc-Lys(Boc)-OH, Fmoc-Gly-OH, Fmoc-Ser(O*t*Bu)-OH and ribosyl building block from Voorneveld *et al.*[1] After HPLC purification, compound **2** was obtained as an orange powder (1.85 mg, 0.98 µmol, 4.0%). **$^1$H NMR** (600 MHz, $D_2O$ + $CD_3CN$) δ 8.55 (d, *J* = 7.4 Hz, 1H, H-2), 8.28 (d, *J* =

10.6 Hz, 1H, H-8), 8.23 (d, *J* = 1.6 Hz, 0.5H, CF), 8.03 (d, *J* = 1.6 Hz, 0.5H, CF), 7.95 (dd, *J* = 7.7, 1.7 Hz, 0.5H, CF), 7.68 (dd, *J* = 7.8, 1.6 Hz, 0.5H, CF), 7.54 – 7.44 (m, 0.5H, CF) 7.36 (m, 0.5H, CF), 7.03 – 6.94 (m, 2H, CF), 6.91 – 6.83 (m, 2H, CF), 6.80 – 6.71 (m, 2H, CF), 6.12 (dd, *J* = 5.6, 2.4 Hz, 1H, H-1'), 5.49 (d, *J* = 4.6 Hz, 1H, H-1"). **$^{31}$P NMR** δ -10.29, -10.47, -10.59, -10.75. **LC-MS** (10% -> 50% MeCN) Rt = 6.26 min. **HRMS** [$C_{75}H_{99}N_{15}O_{32}P_2S + 2H^+$] found: 908.7956, calculated: 908.7961.

**CF-Arg-Pro-Thr-Pro-Cys(5-O-adenosine-diphosphate-α-D-ribosyl)-Ala-Pro-Leu-Ala-OH (3)**

The general procedures were followed as described to 25 µmol Tentagel® S AC resin unless stated otherwise. The amino acids used were Fmoc-Arg(Pbf)-OH, Fmoc-Pro-OH, Fmoc-Thr(O*t*Bu)-OH, Fmoc-Leu-OH, Fmoc-Ala-OH and ribosyl building block **16**. After HPLC purification, compound **3** was obtained as an orange powder (3.86 mg, 2.07 µmol, 8.3%). **$^{1}$H NMR** δ 8.56 (m, 1H, H-2), 8.38 (s, 1H, CF), 8.13 (m, 1H, H-8), 7.45 (m, 1H, CF), 7.14 (m, 2H, CF), 6.92 (d, *J* = 11.7 Hz, 2H, CF), 6.82 (dd, *J* = 15.8, 9.2 Hz, 2H, CF), 6.11 (m, 1H, H-1'), 5.53 (d, *J* = 4.8 Hz, 1H, H-1").**$^{31}$P NMR** (202 MHz, $D_2O$) δ -10.98, -11.08, -11.19, -11.22, -11.24, -11.32. δ **LC-MS** (10% -> 50% MeCN) Rt = 5.02 min. **HRMS** [$C_{76}H_{99}N_{17}O_{30}P_2S + 2H^+$] found: 912.8037, calculated: 912.8043.

**CF-Arg-Pro-Thr-Pro-Cys-Ala-Pro-Leu-Ala-Glu-Cys(5-O-adenosine-diphosphate-α-D-ribosyl)-Lys-Gly-Ser-Leu-OH (4)**

The general procedures were followed as described to 50 µmol Tentagel® S AC resin unless stated otherwise. The amino acids used were Fmoc-Arg(Pbf)-OH, Fmoc-Pro-OH, Fmoc-Thr(O*t*Bu)-OH, Fmoc-Leu-OH, Fmoc-Ala-OH, Fmoc-Glu(O*t*Bu)-OH, Fmoc-Lys(Boc)-OH, Fmoc-Gly-OH, Fmoc-Ser(O*t*Bu)-OH, Fmoc-Cys(Trt)-OH and ribosyl building block **16**. After HPLC purification, compound **4** was obtained as an orange powder (2.4 mg, 0.98 µmol, 2.0%).**$^{1}$H NMR** (500 MHz, $D_2O$ + $CD_3CN$) δ 8.62 (s, 2H, H-2), 8.47 – 8.44 (m, 1H, CF), 8.38 – 8.32 (m, 2H, H-8), 8.19 (t, *J* = 6.6 Hz, 1H, CF), 7.55 – 7.49 (m, 1H, CF), 7.22 (m, 2H, CF), 7.04 – 6.93 (m, 2H, CF), 6.92 – 6.83 (m, 2H, CF), 6.20 (m, 2H, H-1'), 5.72 – 5.63 (m, 0.2H, H-1"), 5.58 (d, *J* = 5.2 Hz, 1.3H, H-1"), 5.18 – 5.13 (m, 0.1H, H-1"), 5.11 (d, *J* = 5.4 Hz, 0.4H, H-1"). **$^{31}$P NMR** (202 MHz, $CH_3CN$+$D_2O$) δ 1.08, -4.03, -10.33- -10.42, -10.58, -10.69. **LC-MS** (10% -> 90% MeCN) Rt = 3.70 min. **HRMS**

**CF-Arg-Pro-Thr-Pro-Cys(5-O-adenosine-diphosphate-α-D-ribosyl)-Ala-Pro-Leu-Ala-Glu-Cys-Lys-Gly-Ser-Leu-OH (5)**

The general procedures were followed as described to 50 µmol Tentagel® S AC resin unless stated otherwise. The amino acids used were Fmoc-Arg(Pbf)-OH, Fmoc-Pro-OH, Fmoc-Thr(O*t*Bu)-OH, Fmoc-Leu-OH, Fmoc-Ala-OH, Fmoc-Glu(O*t*Bu)-OH, Fmoc-Lys(Boc)-OH, Fmoc-Gly-OH, Fmoc-Ser(O*t*Bu)-OH, Fmoc-Cys(Trt)-OH and ribosyl building block **16**. After HPLC purification, compound **4** was obtained as an orange powder (2.8 mg, 1.1 µmol, 2.3%). **$^{1}$H NMR** (500 MHz, $D_2O$ +$CD_{3C}N$) δ 9.03 (s, 1H ,H-2), 8.92 (s, 1H, CF), 8.77 (s, 1H, H-8), 8.69 (d, *J* = 8.0 Hz, 1H, CF), 7.82 (d, *J* = 8.2 Hz, 1H, CF), 7.27 (s, 2H, CF), 7.18 (d, *J* = 8.9 Hz, 2H, CF), 7.10 (d, *J* = 8.7 Hz, 2H, CF), 6.56 (d, *J* = 5.5 Hz, 1H, H-1'), 5.94 (m, 1H, H-1"). **$^{31}$P NMR** (202 MHz, $D_2O$) δ 3.66, 3.33, -1.65, -7.68, -7,78, -7.94, -7.99, -8.05. **LC-MS** (10% -> 90% MeCN) Rt = 3.72 min. **HRMS**

## List of synthesized peptides

Table S1 Peptides synthesized as a control for the fluorescent polarization assay.

| code | Name | sequence |
|---|---|---|
| **1** | $AR_{280-294}ADPr$ | CF-RPTP**C**(ADPr)APLAE**C**(ADPr)KGSL-OH |
| **2** | $AR_{286-294}ADPr$ | CF-PLAE**C**(ADPr)KGSL-OH |
| **3** | $AR_{280-288}ADPr$ | CF-RPTP**C**(ADPr)APLA-OH |
| **4** | $AR_{280-294}ADPr$ | CF-RPTPCAPLAE**C**(ADPr)KGSL-OH |
| **5** | $AR_{280-294}ADPr$ | CF-RPTP**C**(ADPr)APLAECKGSL-OH |
| **6** | $AR_{280-294}$ | CF-RPTPCAPLAECKGSL-OH |

Amino acids are given in the one-letter code. Peptides are synthesized according to the aforementioned general protocols for peptide synthesis and installation of CF. ADP-ribosylated amino acid residues are highlighted. Abbreviations CF = (5)-carboxy fluorescein (CF).

# HP-LC/MS traces of crude MAR-peptides

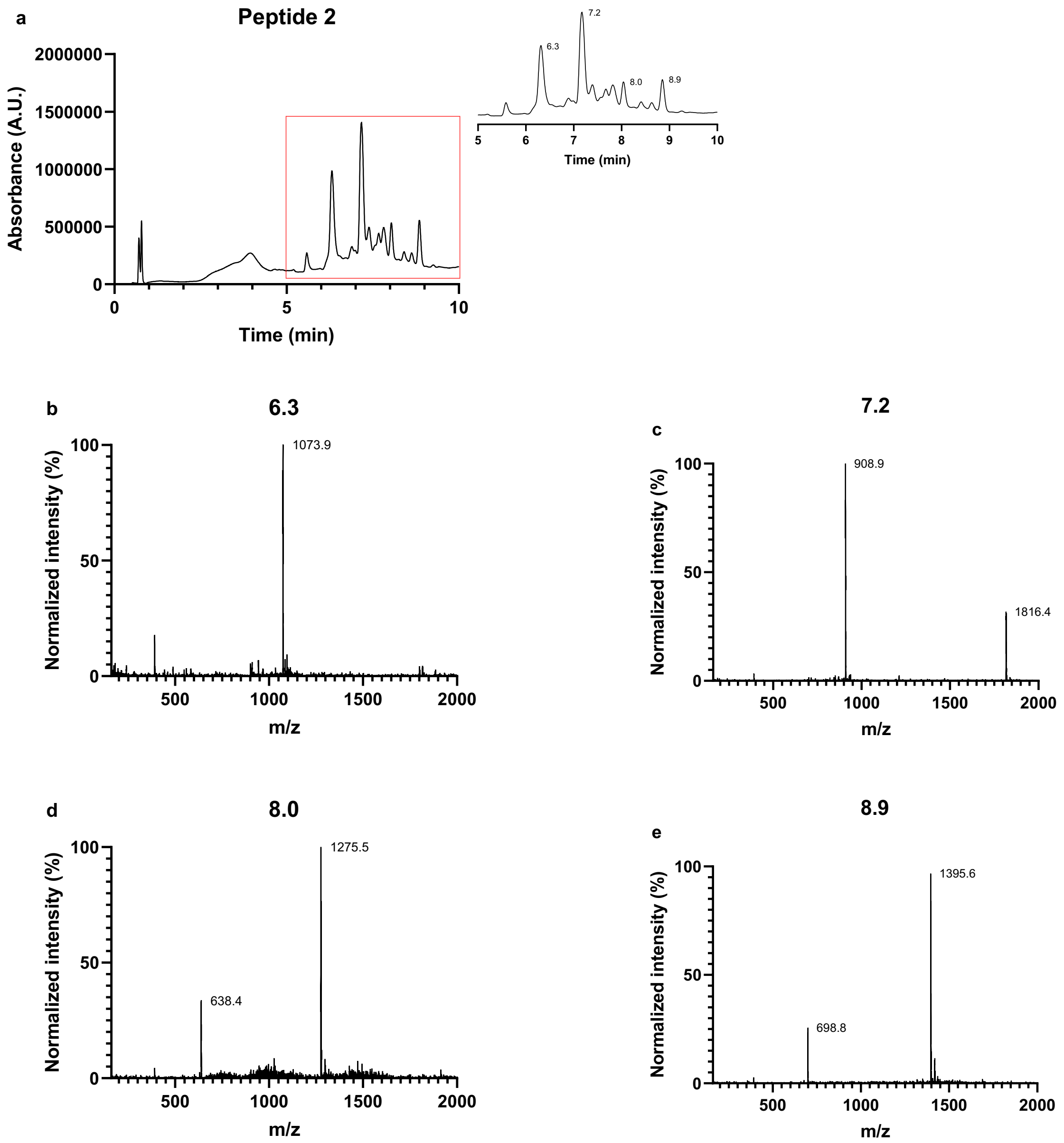


*Figure S1 Analytical HP-LC (linear gradient 10-50% ACN over 12 min.) of crude peptide **2**. a)* Total scan chromatogram of peptide **2**, relevant peaks are highlighted and retention times are given above. *b) ESI mass spectrum of peak with Rt= 6.3 min. corresponding to peptide **2** +AMP, expected mass: 2144.6 Da, deconvoluted mass: 2145.8 Da. c) ESI mass spectrum of peak with Rt= 7.2 min. corresponding to peptide **2**, expected mass: 1815.6 Da, deconvoluted mass: 1815.4 Da. d) ESI mass spectrum of peak with Rt= 8.0 min. corresponding to peptide **2** -ADPr, expected mass: 1274.5 Da, deconvoluted mass: 1274.5 Da. e) ESI mass spectrum of peak with Rt= 8.9 min. corresponding to peptide **2** with PMB migration to thiol, expected mass: 1394.6 Da, deconvoluted mass: 1394.6 Da.*

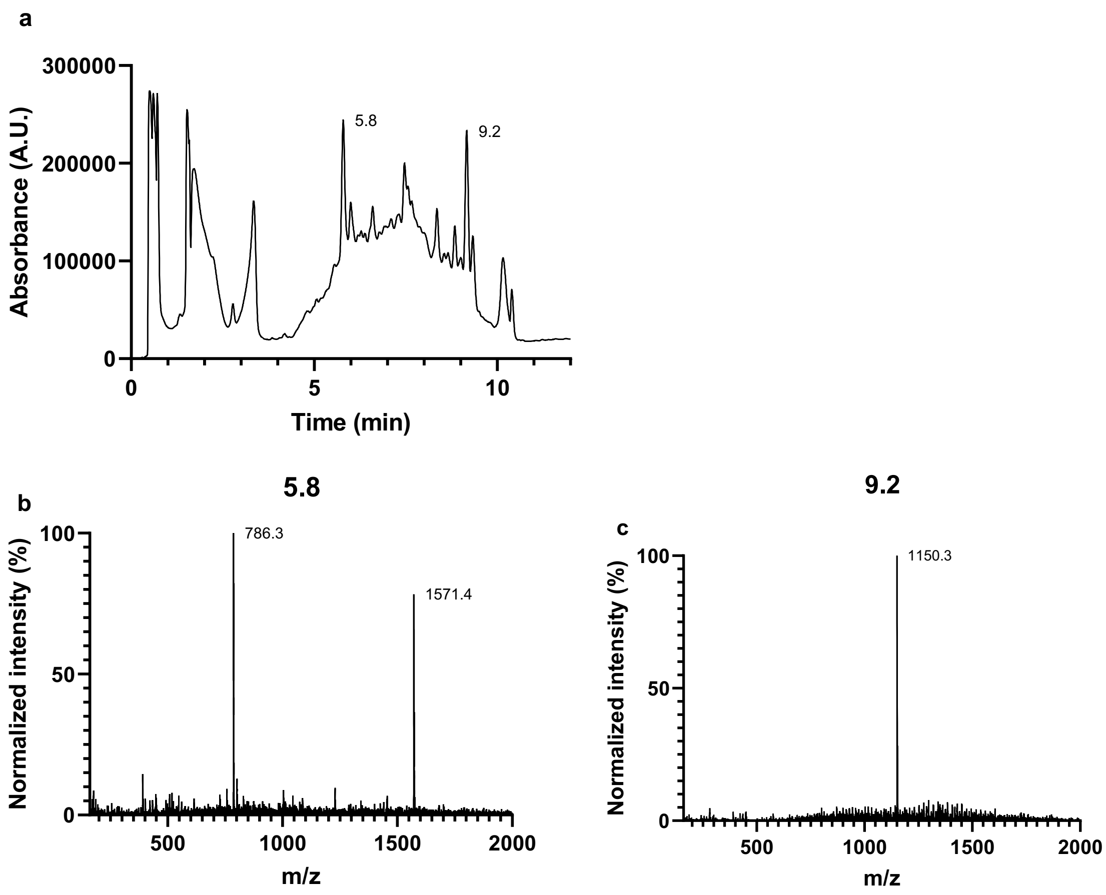


*Figure S2 Analytical HP-LC (linear gradient 10-50% ACN over 12 min.) of crude tandem ADP-ribosylated peptide, synthesized via the procedure from Voorneveld et al.*[1] *a)* Total scan chromatogram of the peptide, relevant peaks are highlighted and retention times are given above. *b+c) ESI mass spectrum of peaks with Rt= 5.8 min and 9.2 min.*

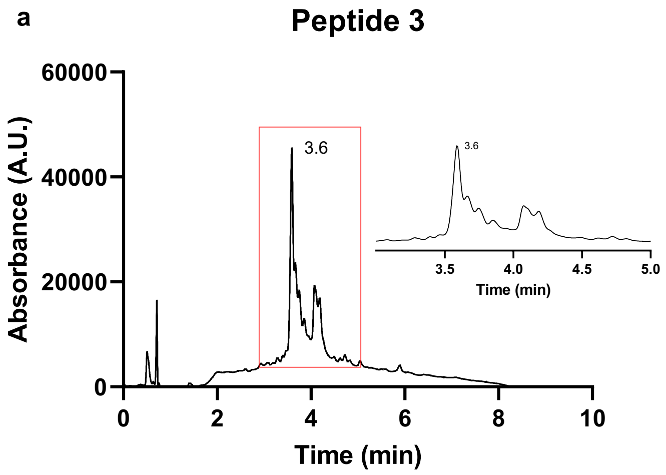


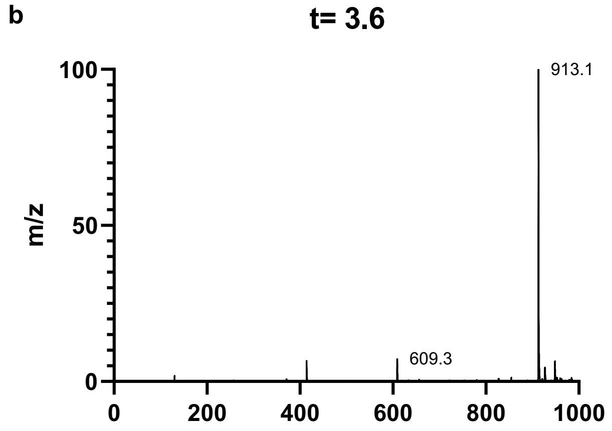


*Figure S3 Analytical HP-LC (linear gradient 10-90% ACN over 12 min.) of crude peptide **3**. a)* Total scan chromatogram of peptide **3**, relevant peaks are highlighted and retention times are given above. *b) ESI mass spectrum of peak with Rt= 3.6 min. corresponding to peptide **3**, expected mass: 1823.6 Da, deconvoluted mass: 1824.2 Da.*

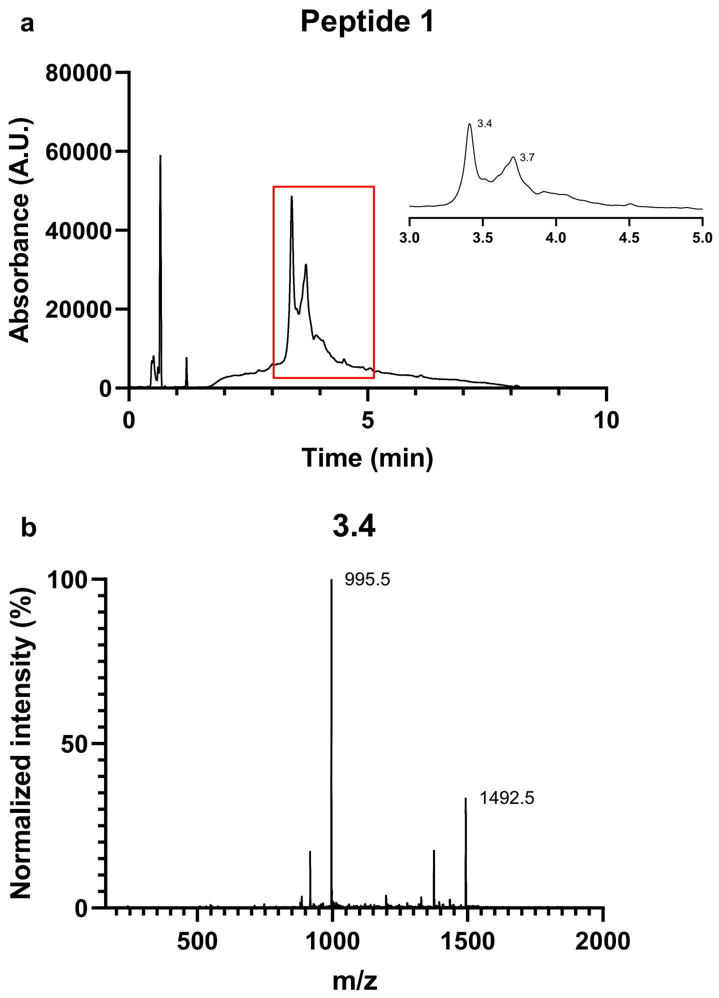


*Figure S4 Analytical HP-LC (linear gradient 10-90% ACN over 12 min.) of crude peptide **1**. a)* Total scan chromatogram of peptide **1**, relevant peaks are highlighted and retention times are given above. *b) ESI mass spectrum of peak with Rt= 3.4 min. corresponding to peptide **1**, expected mass: 2981.9 Da, deconvoluted mass: 2983.0 Da.*

# HP-LC/MS traces of purified MAR-peptides

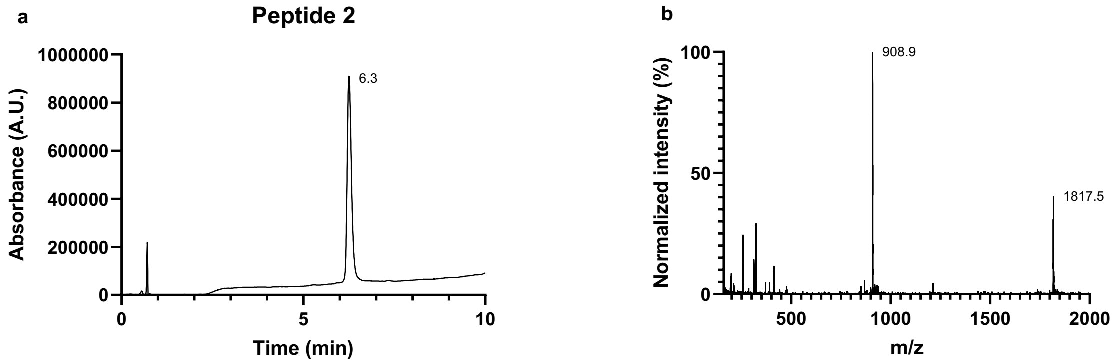


*Figure S5 Analytical HP-LC (linear gradient 10-50% ACN over 12 min.) of pure peptide **2**. a) Total scan chromatogram of peptide **2**, relevant peaks are highlighted and retention time is given above. b) ESI mass spectrum of peak with Rt= 6.3 min. expected mass: 1816.6 Da, deconvoluted mass: 1816.5 Da.*

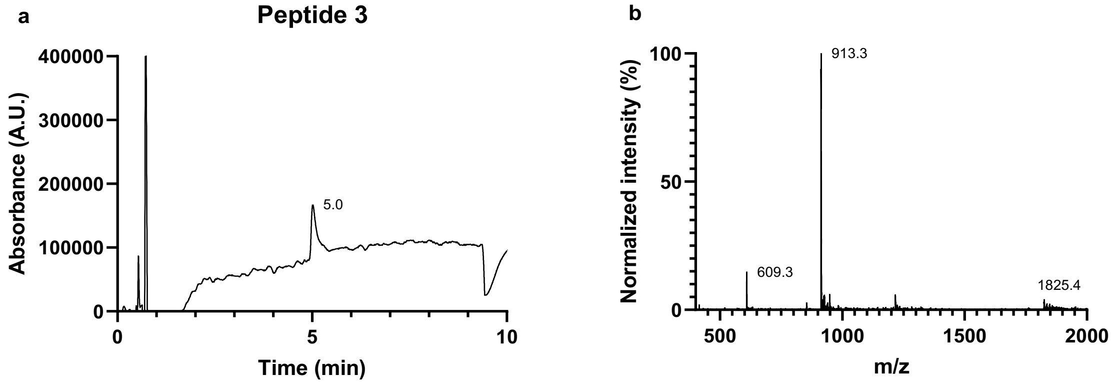


*Figure S6 Analytical HP-LC (linear gradient 10-50% ACN over 12 min.) of pure peptide **3**. a) Total scan chromatogram of peptide **3**, relevant peaks are highlighted and retention time is given above. b) ESI mass spectrum of peak with Rt= 5.0 min. expected mass: 1823.6 Da, deconvoluted mass: 1824.4 Da.*

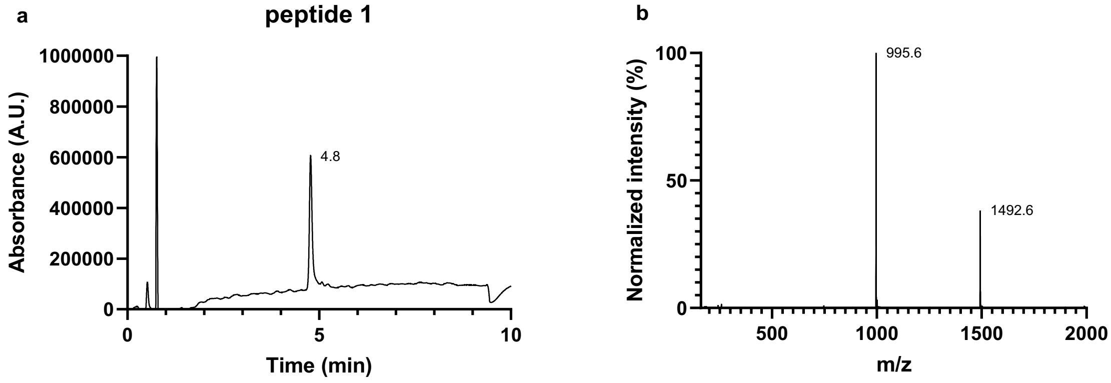


*Figure S7 Analytical HP-LC (linear gradient 10-50% ACN over 12 min.) of pure peptide **1**. a) Total scan chromatogram of peptide **1**, relevant peaks are highlighted and retention time is given above. b) ESI mass spectrum of peak with Rt= 4.8 min. expected mass: 2981.9Da, deconvoluted mass: 2983.2 Da.*

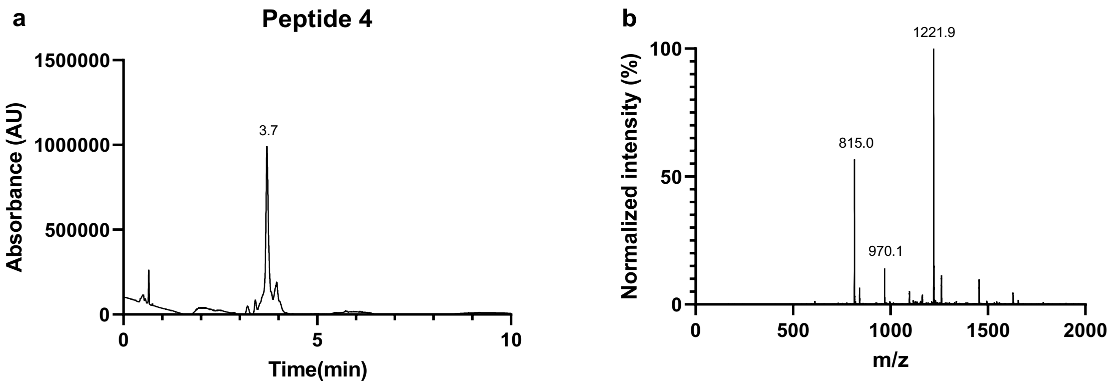


*Figure S8 Analytical HP-LC (linear gradient 10-90% ACN over 12 min.) of pure peptide **4**. a) Total scan chromatogram of peptide **4**, relevant peaks are highlighted and retention time is given above. b) ESI mass spectrum of peak with Rt= 3.7 min. expected mass: 2440.9 Da, deconvoluted mass: 2441.8 Da.*

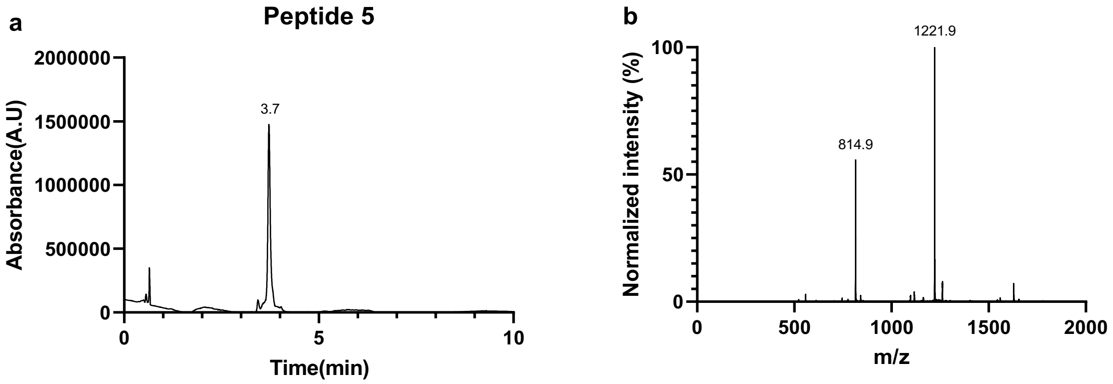


*Figure S9 Analytical HP-LC (linear gradient 10-90% ACN over 12 min.) of pure peptide **5**. a) Total scan chromatogram of peptide **5**, relevant peaks are highlighted and retention time is given above. b) ESI mass spectrum of peak with Rt= 3.7 min. expected mass: 2440.9 Da, deconvoluted mass: 2441.8 Da.*

# SDS Page gels:

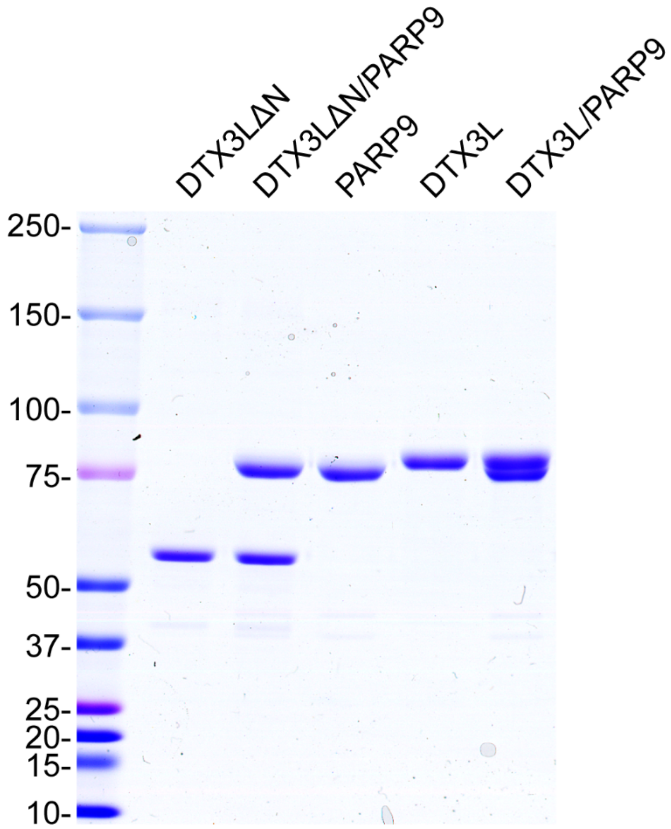


*Figure S10 DTX3L and PARP9 proteins used for the fluorescence polarization assay. Equimolar amounts of recombinant proteins (15 pmol each) were seperated by 4-20% SDS PAGE followed with Coomassie Brilliant Blue R-250 staining.*

# NMR spectra:

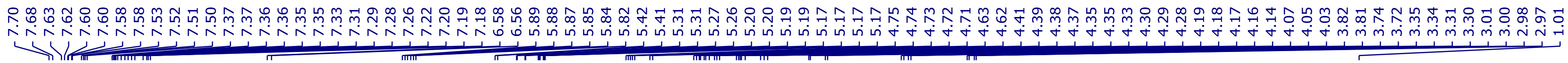


1-S-5-O-((tert-butyl)-diphenylsilyl)α-D-ribosyl)-N-fluorenylmethoxycarbonyl cysteine allyl ester (17)

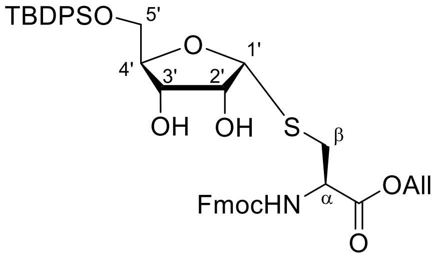


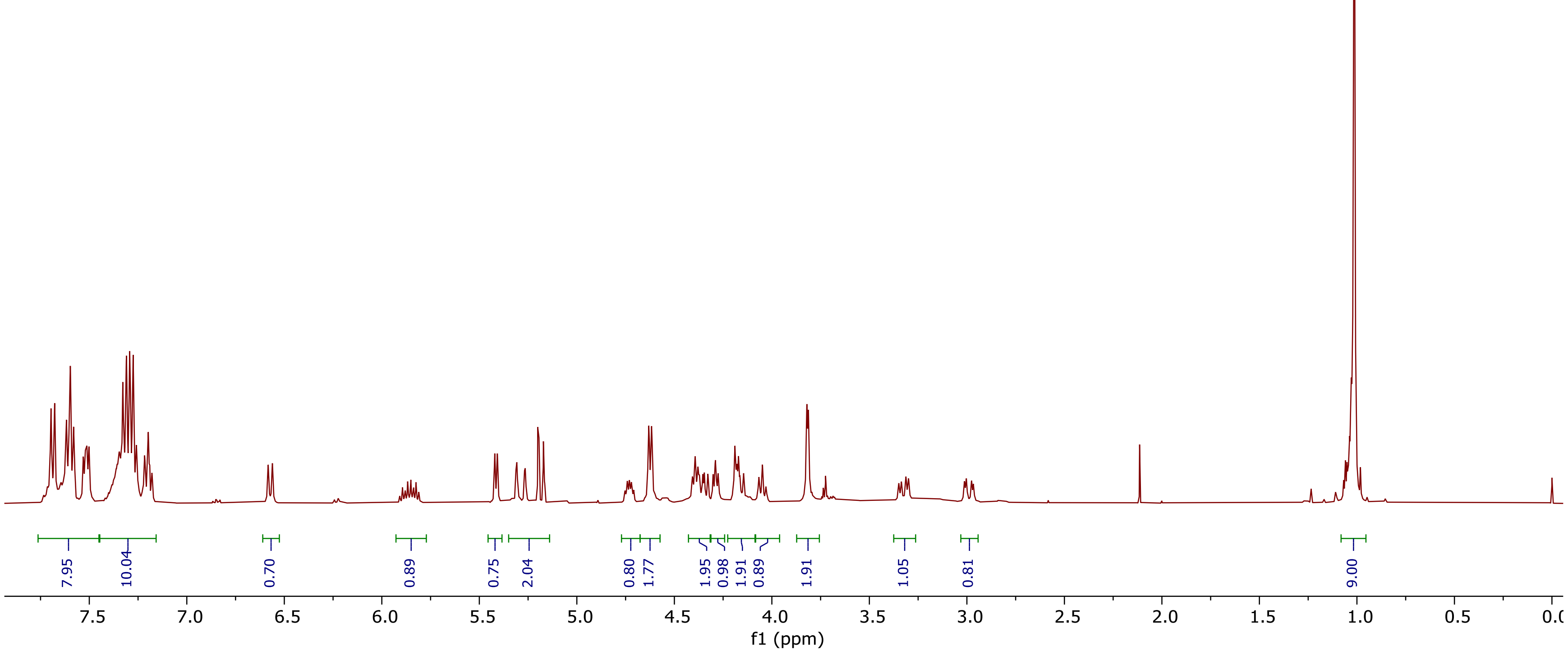

1-S-5-O-((tert-butyl)-diphenylsilyl)α-D-ribosyl)-N-fluorenylmethoxycarbonyl cysteine allyl ester (17)

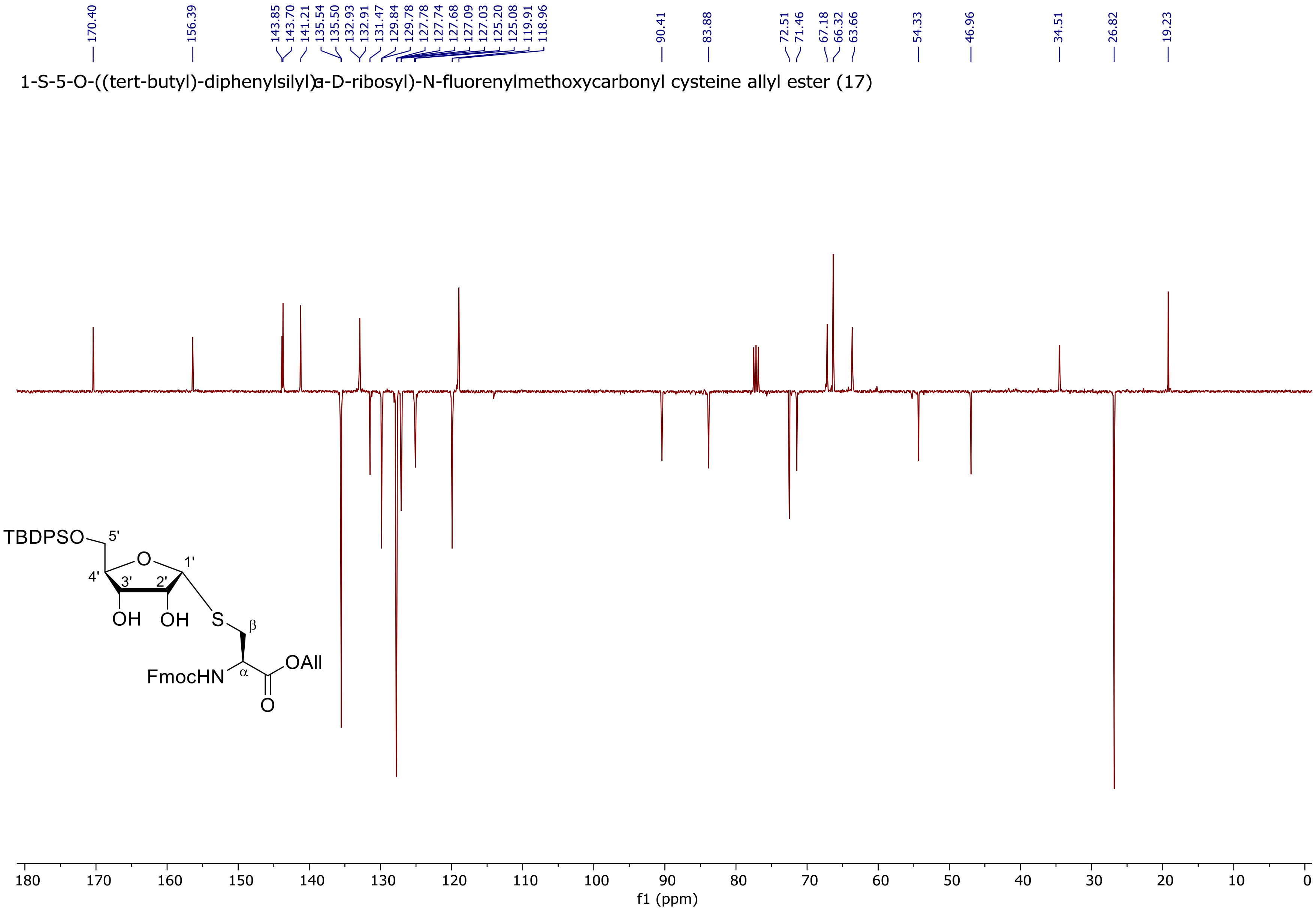

1-S-2,3-bis-O-(tert-butyloxycarbonyl)-5-O-((tert-butyl)-diphenylsilyl)-β-D-ribosyl)-N-fluorenylmethoxycarbonyl cysteine allyl ester (28)

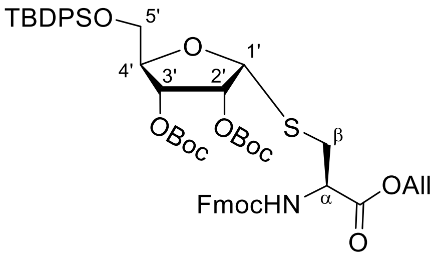


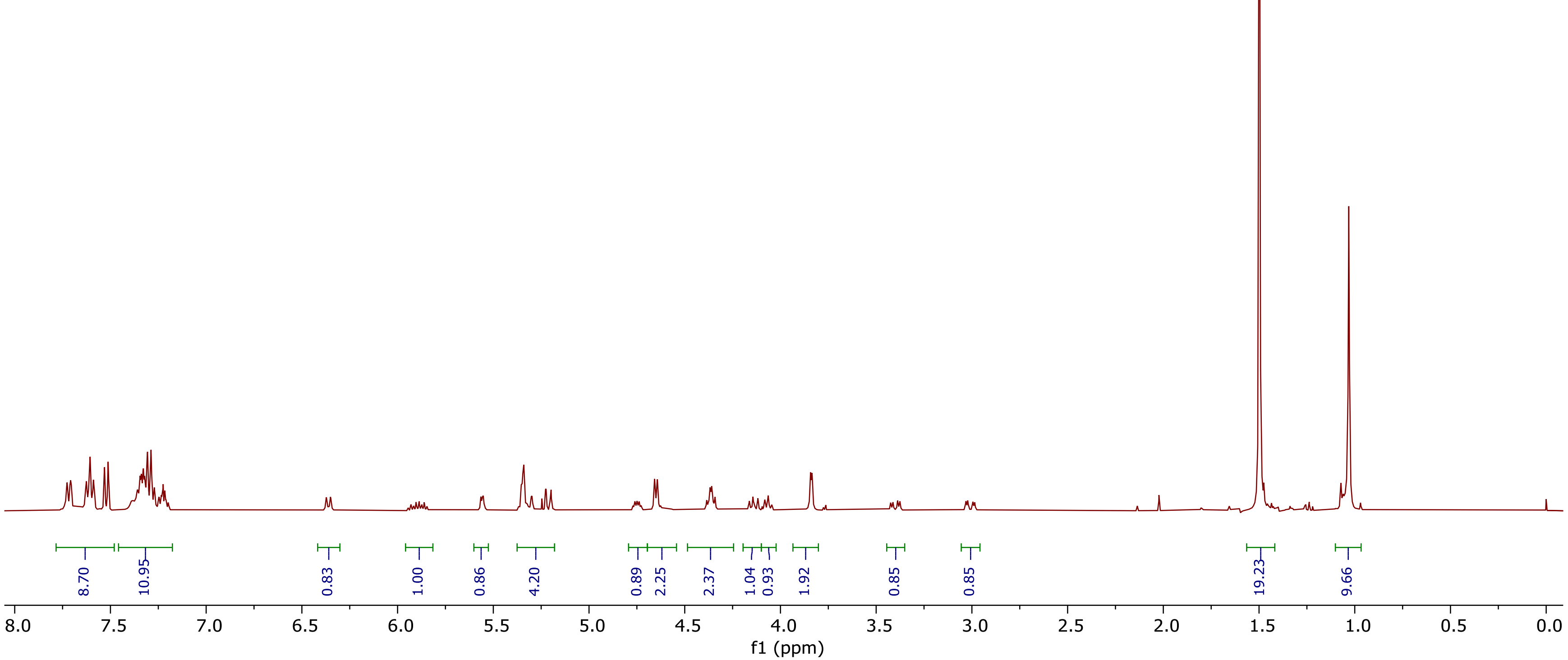

1-S-2,3-bis-O-(tert-butyloxycarbonyl)-5-O-((tert-butyl)-diphenylsilyl)-D-ribosyl)-N-fluorenylmethoxycarbonyl cysteine allyl ester (28)

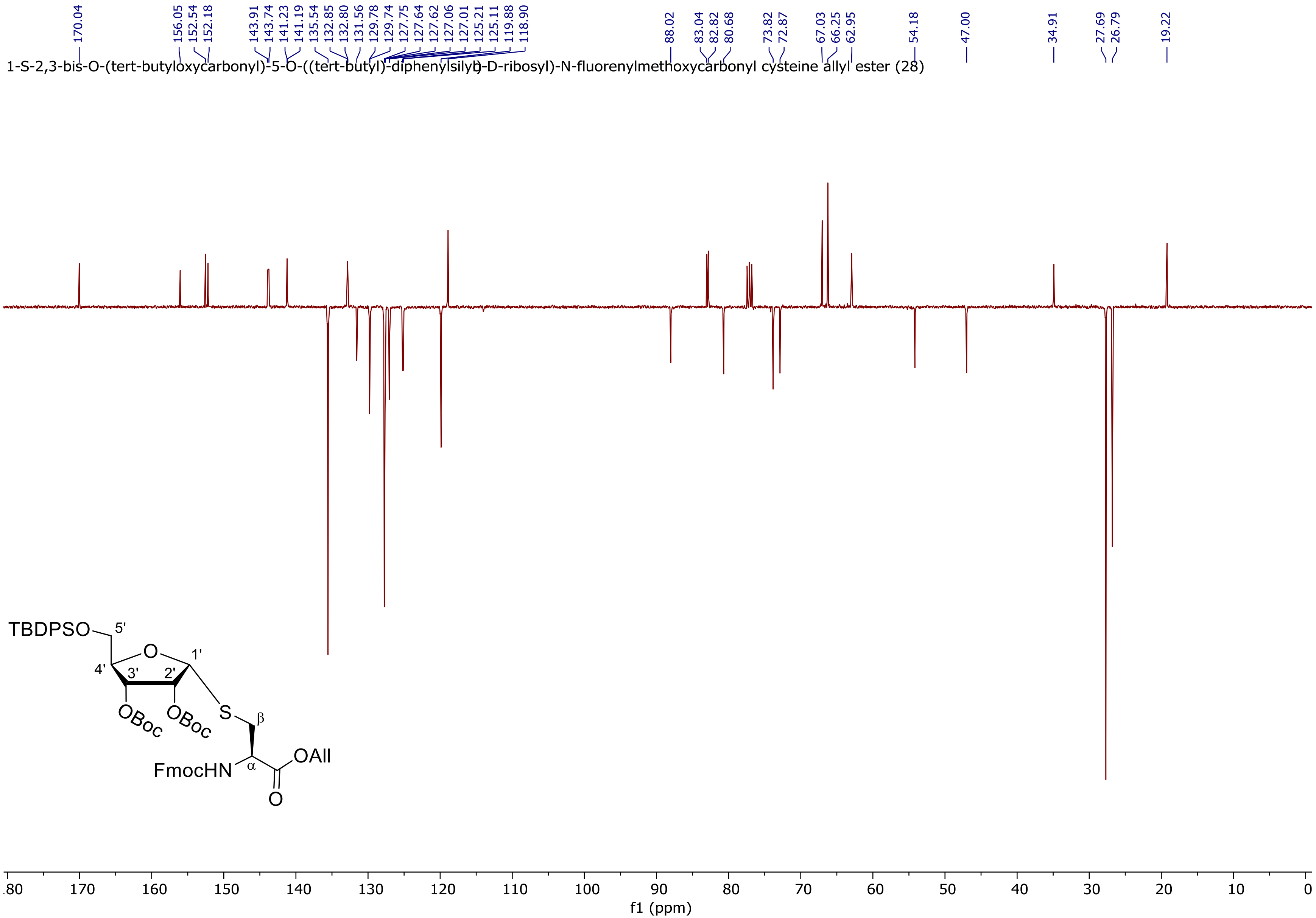

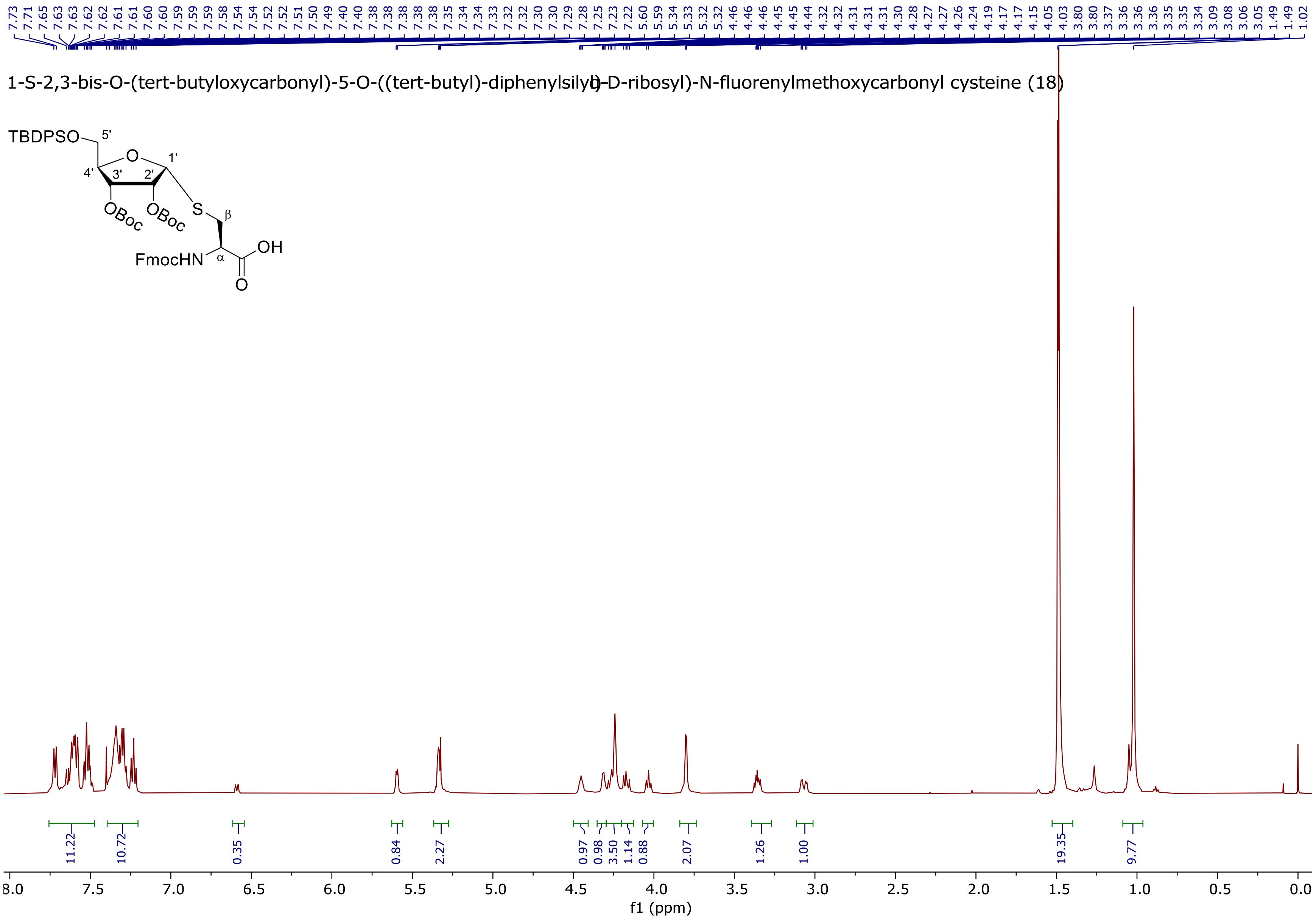
7.73 7.71 7.65 7.63 7.63 7.62 7.62 7.61 7.61 7.60 7.59 7.59 7.59 7.58 7.54 7.54 7.52 7.52 7.51 7.50 7.49 7.40 7.40 7.38 7.38 7.38 7.38 7.38 7.35 7.34 7.34 7.33 7.32 7.32 7.30 7.30 7.29 7.28 7.25 7.23 7.22 5.60 5.59 5.34 5.33 5.32 5.32 4.46 4.46 4.46 4.45 4.45 4.44 4.32 4.32 4.31 4.31 4.30 4.28 4.27 4.27 4.26 4.24 4.19 4.17 4.17 4.15 4.05 4.03 3.80 3.80 3.37 3.36 3.36 3.36 3.35 3.35 3.34 3.09 3.08 3.06 3.05 1.49 1.49 1.02
1-S-2,3-bis-O-(tert-butyloxycarbonyl)-5-O-((tert-butyl)-diphenylsilyl)-D-ribosyl)-N-fluorenylmethoxycarbonyl cysteine (18)
TBDPSO
5'
O
1'
4'
3'
2'
OBoc
OBoc
S
β
FmocHN
α
OH
O
11.22
10.72
0.35
0.84
2.27
0.97
0.98
3.50
1.14
0.88
2.07
1.26
1.00
19.35
9.77
8.0 7.5 7.0 6.5 6.0 5.5 5.0 4.5 4.0 3.5 3.0 2.5 2.0 1.5 1.0 0.5 0.0
f1 (ppm)

1-S-2,3-bis-O-(tert-butyloxycarbonyl)-5-O-((tert-butyl)-diphenylsilyl)-β-D-ribosyl)-N-fluorenylmethoxycarbonyl cysteine (18)

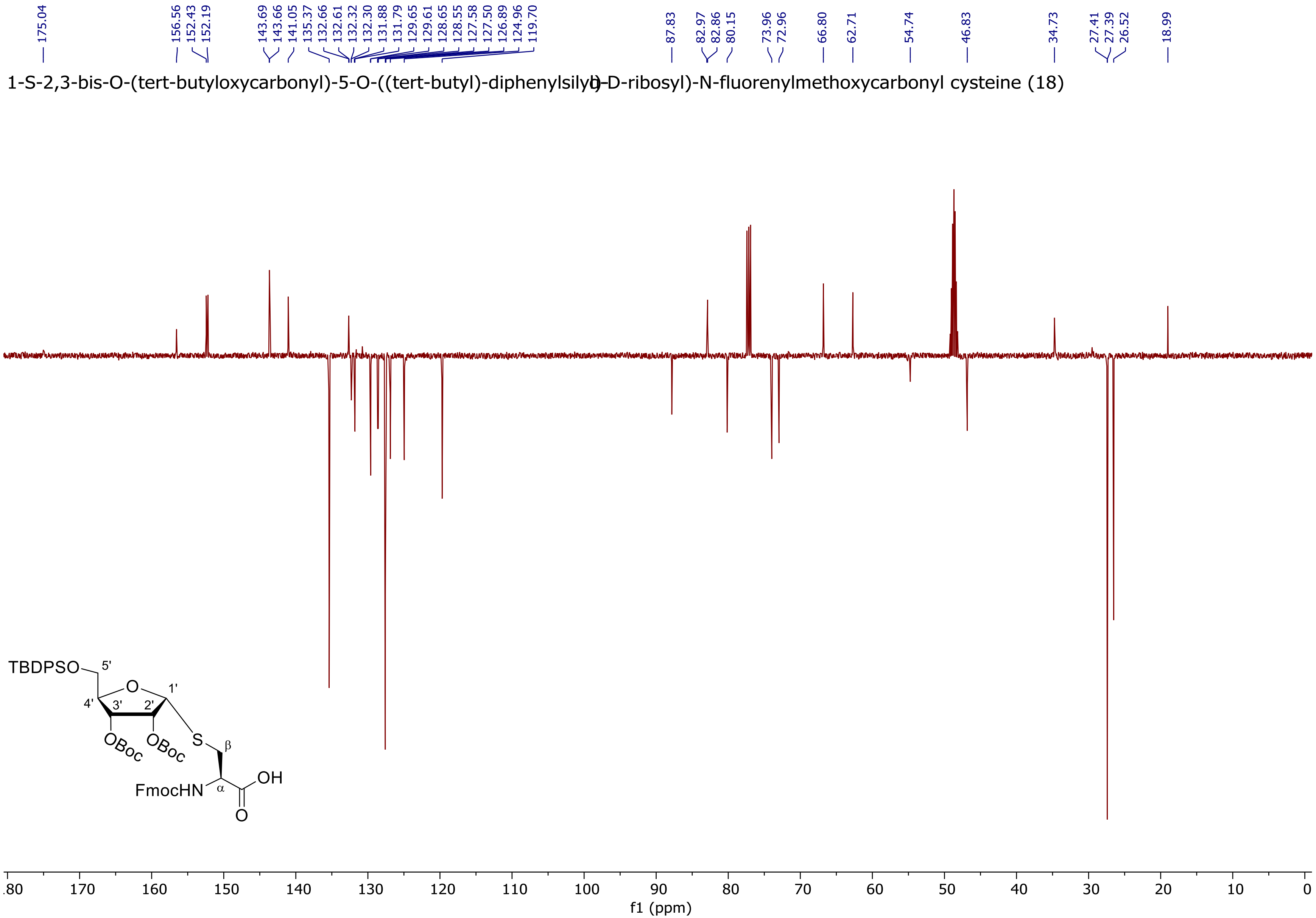

CF-Pro-Leu-Ala-Glu-Cys(5-O-adenosine-diphosphate-α-D-ribosyl)-Lys-Gly-Ser-Leu-OH (2)

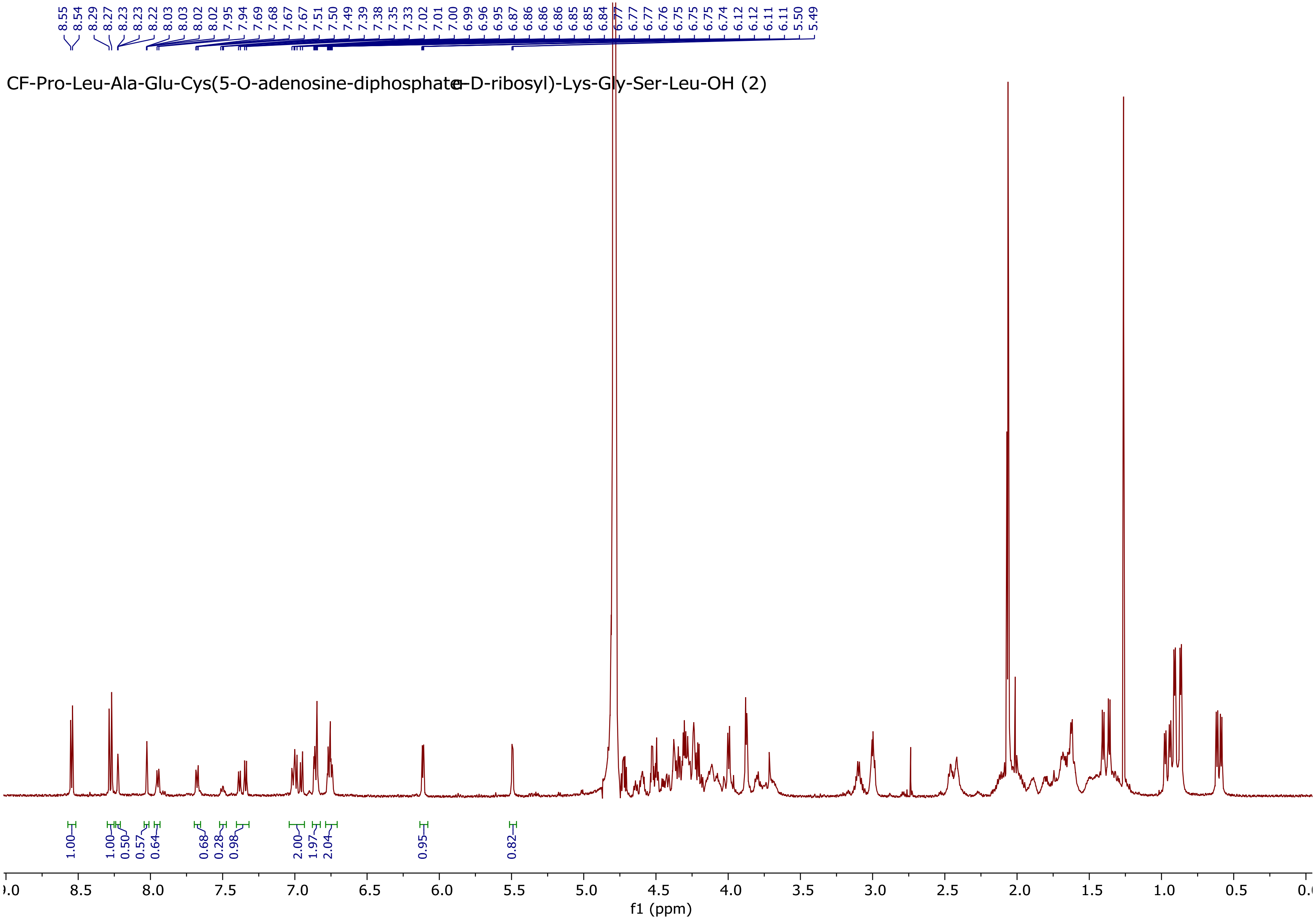

CF-Pro-Leu-Ala-Glu-Cys(5-O-adenosine-diphosphate-α-D-ribosyl)-Lys-Gly-Ser-Leu-OH (2)

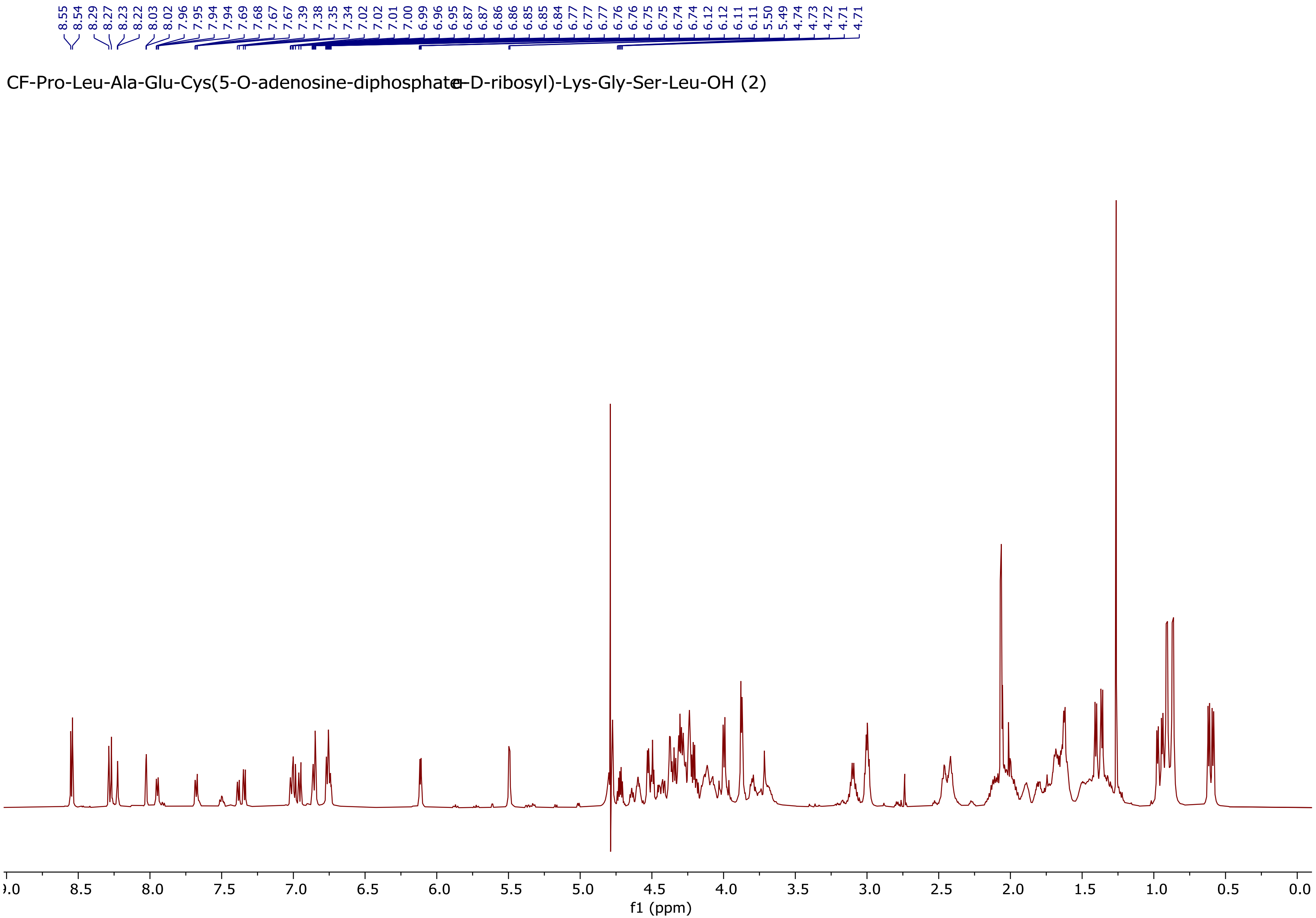

CF-Pro-Leu-Ala-Glu-Cys(5-O-adenosine-diphosphate-α-D-ribosyl)-Lys-Gly-Ser-Leu-OH (2)

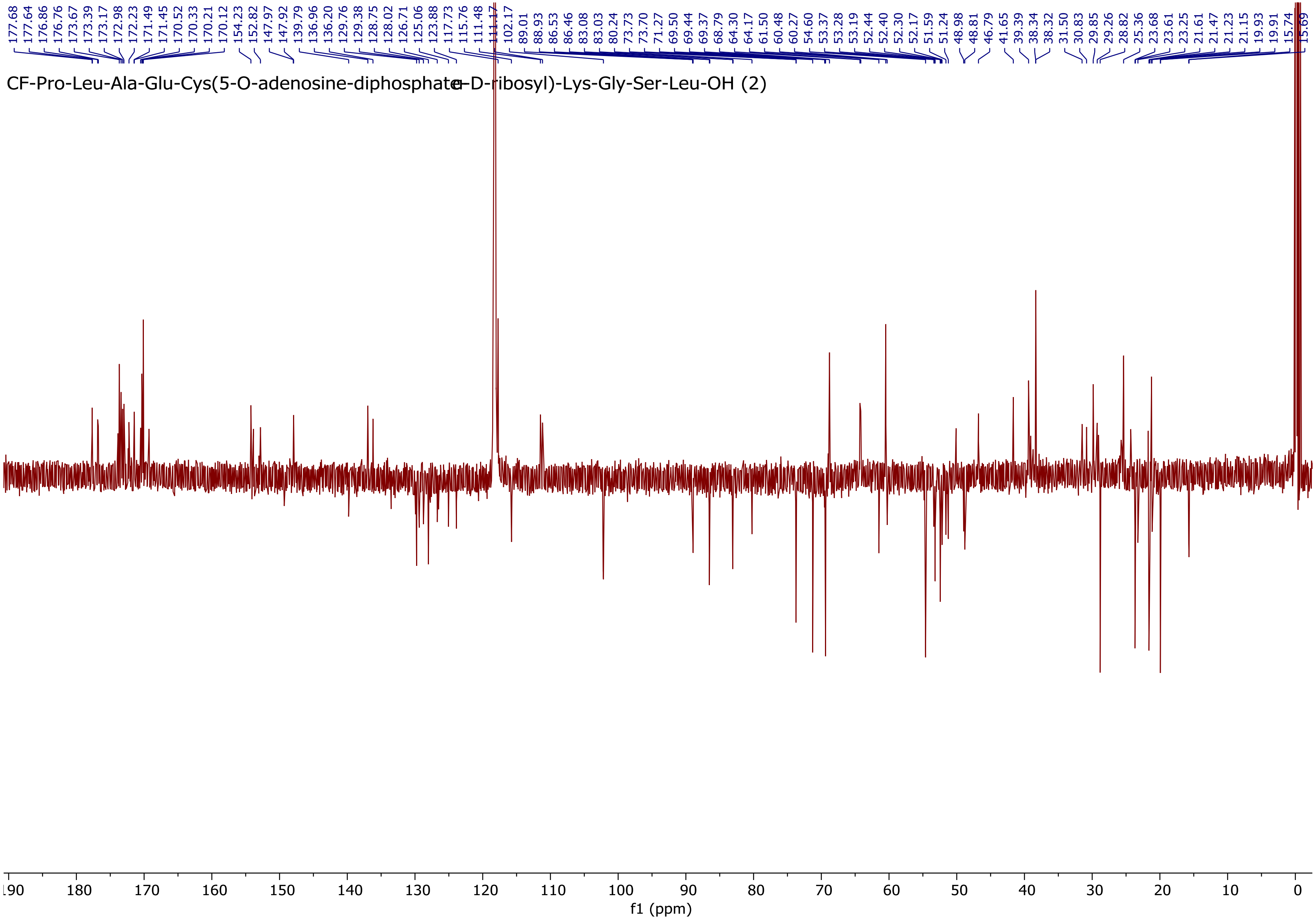

CF-Pro-Leu-Ala-Glu-Cys(5-O-adenosine-diphosphate-α-D-ribosyl)-Lys-Gly-Ser-Leu-OH (2)

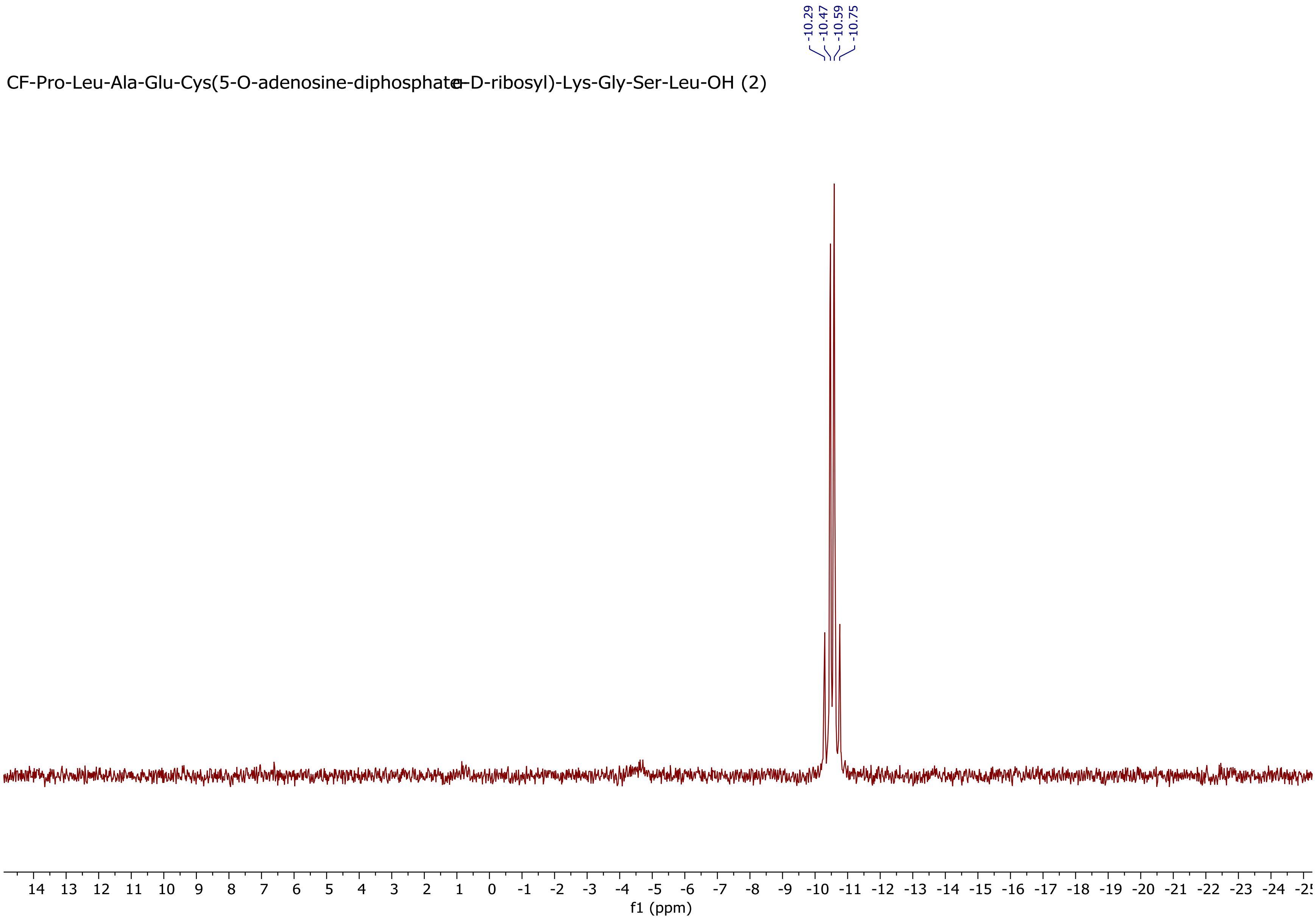

CF-Arg-Pro-Thr-Pro-Cys(5-O-adenosine-diphosphate-α-D-ribosyl)-Ala-Pro-Leu-Ala-OH (3)

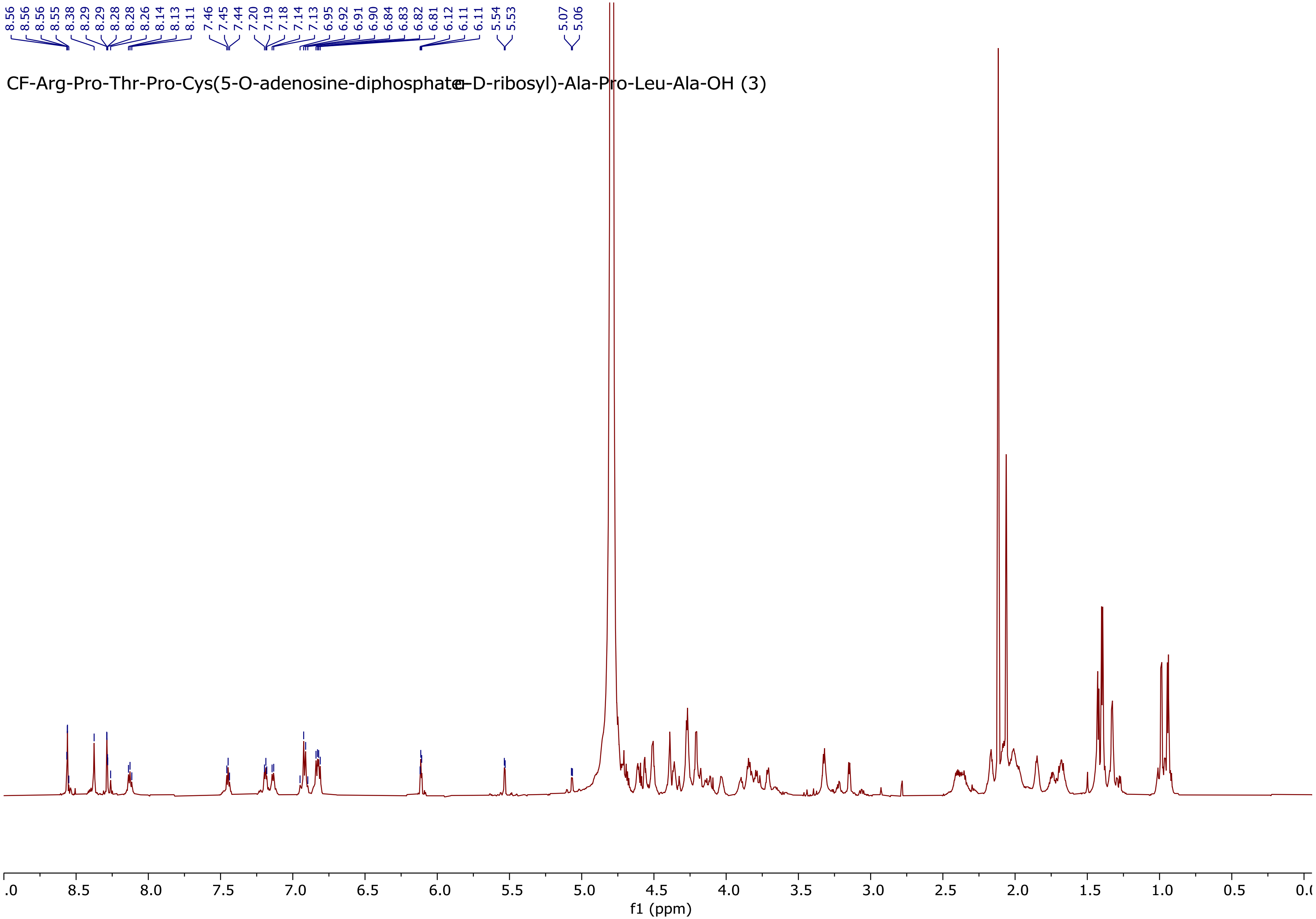

CF-Arg-Pro-Thr-Pro-Cys(5-O-adenosine-diphosphate-α-D-ribosyl)-Ala-Pro-Leu-Ala-OH (3)

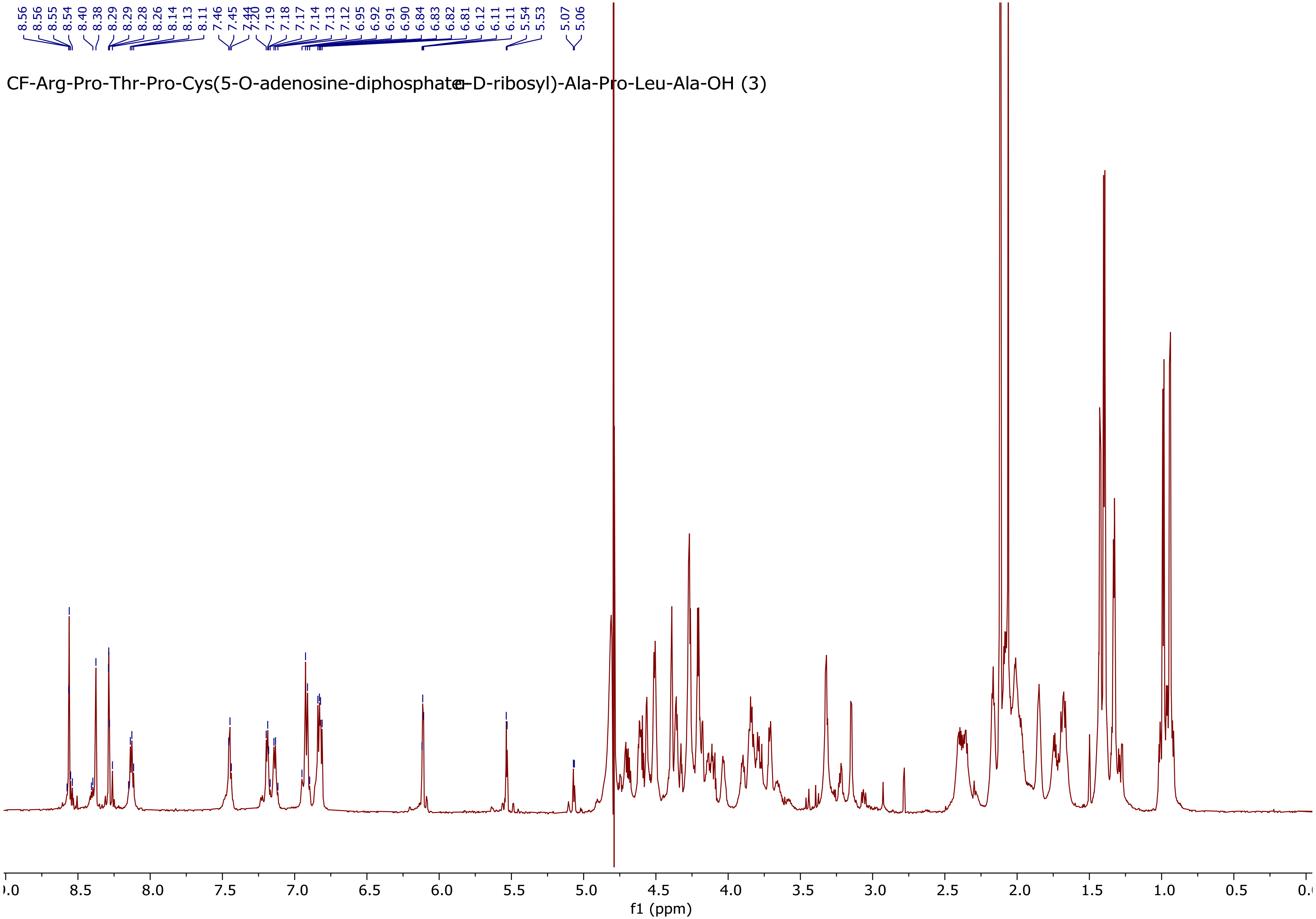

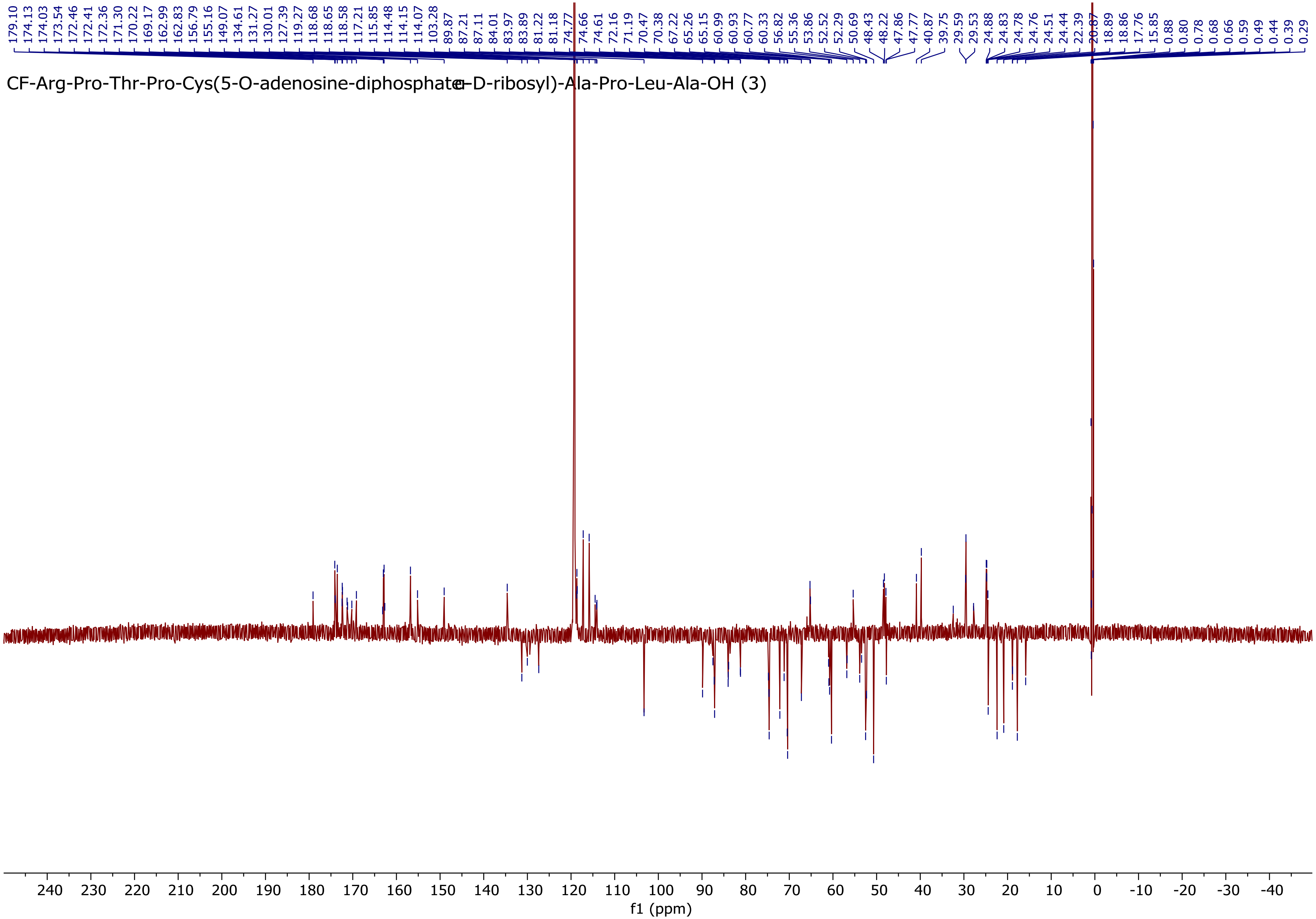


CF-Arg-Pro-Thr-Pro-Cys(5-O-adenosine-diphosphate-α-D-ribosyl)-Ala-Pro-Leu-Ala-OH (3)

CF-Arg-Pro-Thr-Pro-Cys(5-O-adenosine-diphosphate-α-D-ribosyl)-Ala-Pro-Leu-Ala-OH (3)

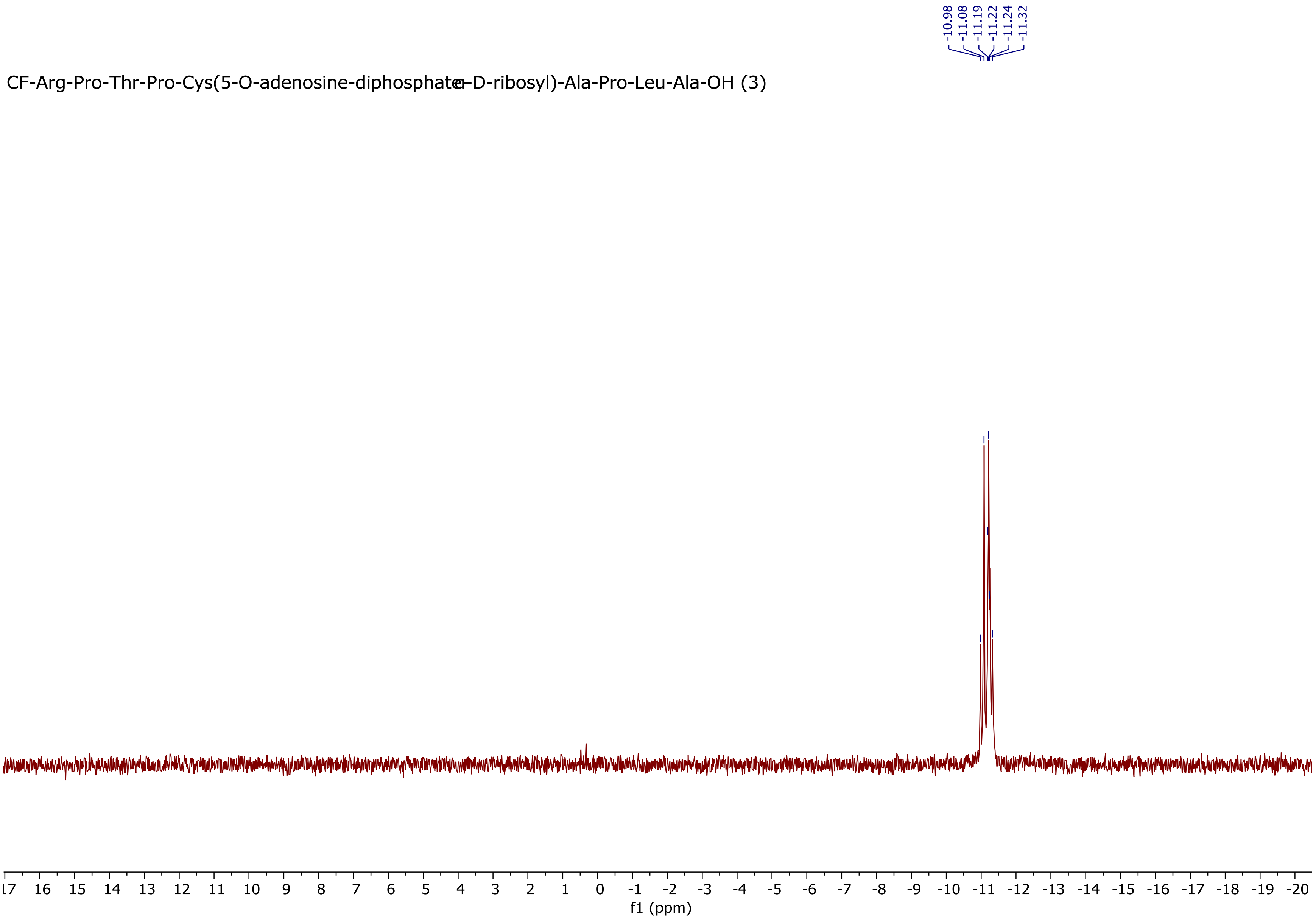

CF-Arg-Pro-Thr-Pro-Cys(5-O-adenosine-diphosphate-α-D-ribosyl)-Ala-Pro-Leu-Ala-Glu-Cys(5-O-adenosine-diphosphate-α-D-ribosyl)
-Lys-Gly-Ser-Leu-OH (1)

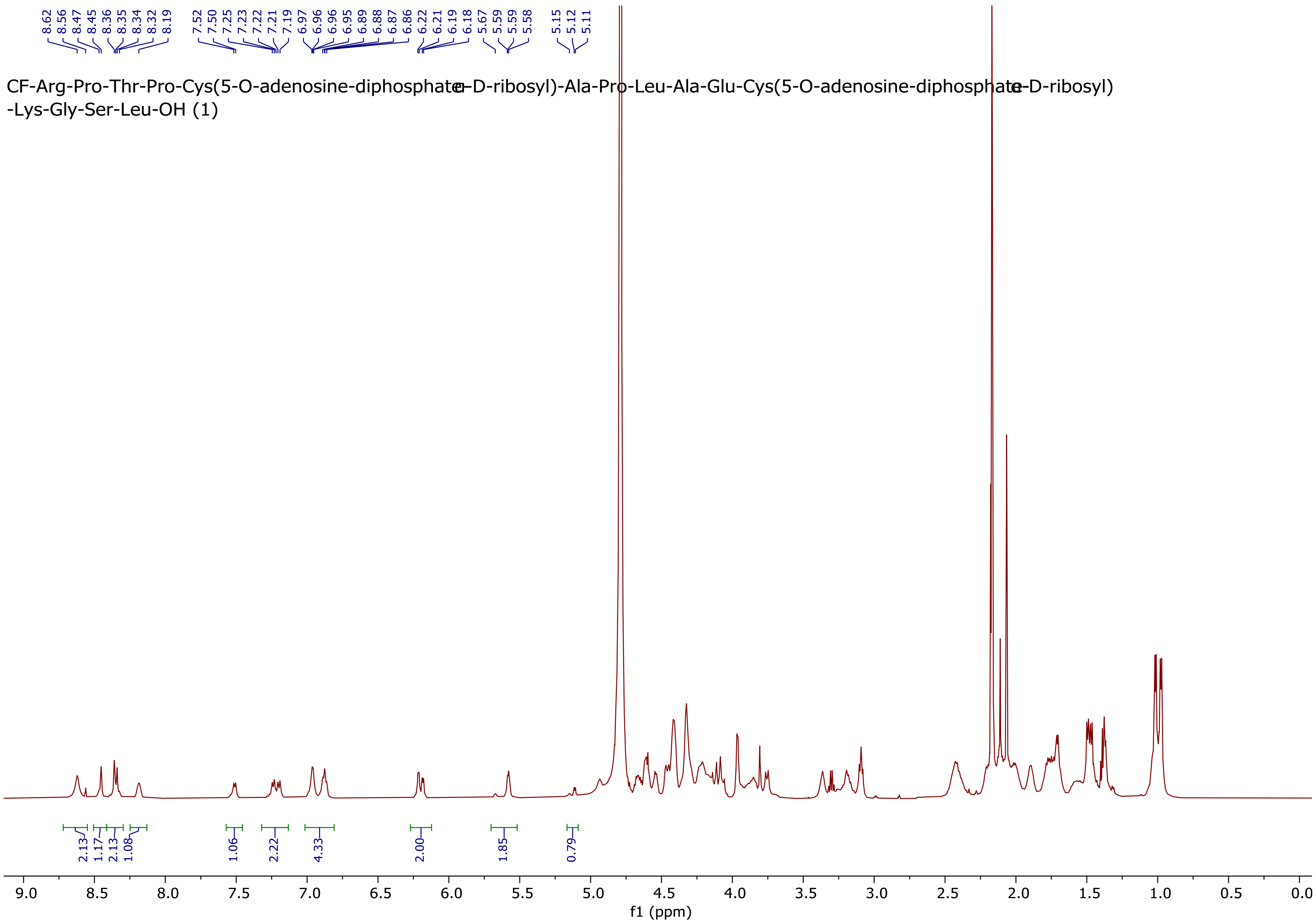

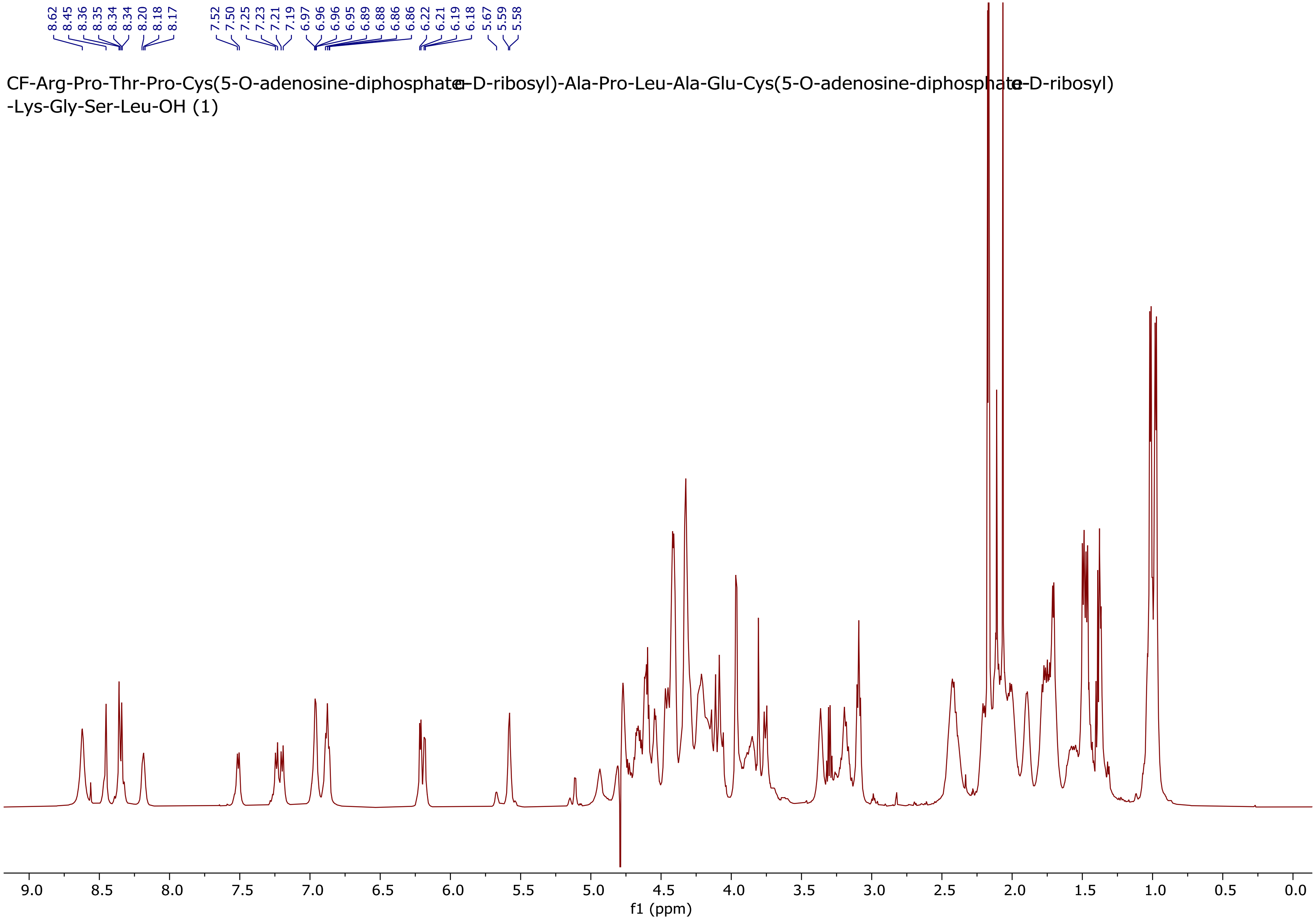


CF-Arg-Pro-Thr-Pro-Cys(5-O-adenosine-diphosphate-α-D-ribosyl)-Ala-Pro-Leu-Ala-Glu-Cys(5-O-adenosine-diphosphate-α-D-ribosyl)
-Lys-Gly-Ser-Leu-OH (1)

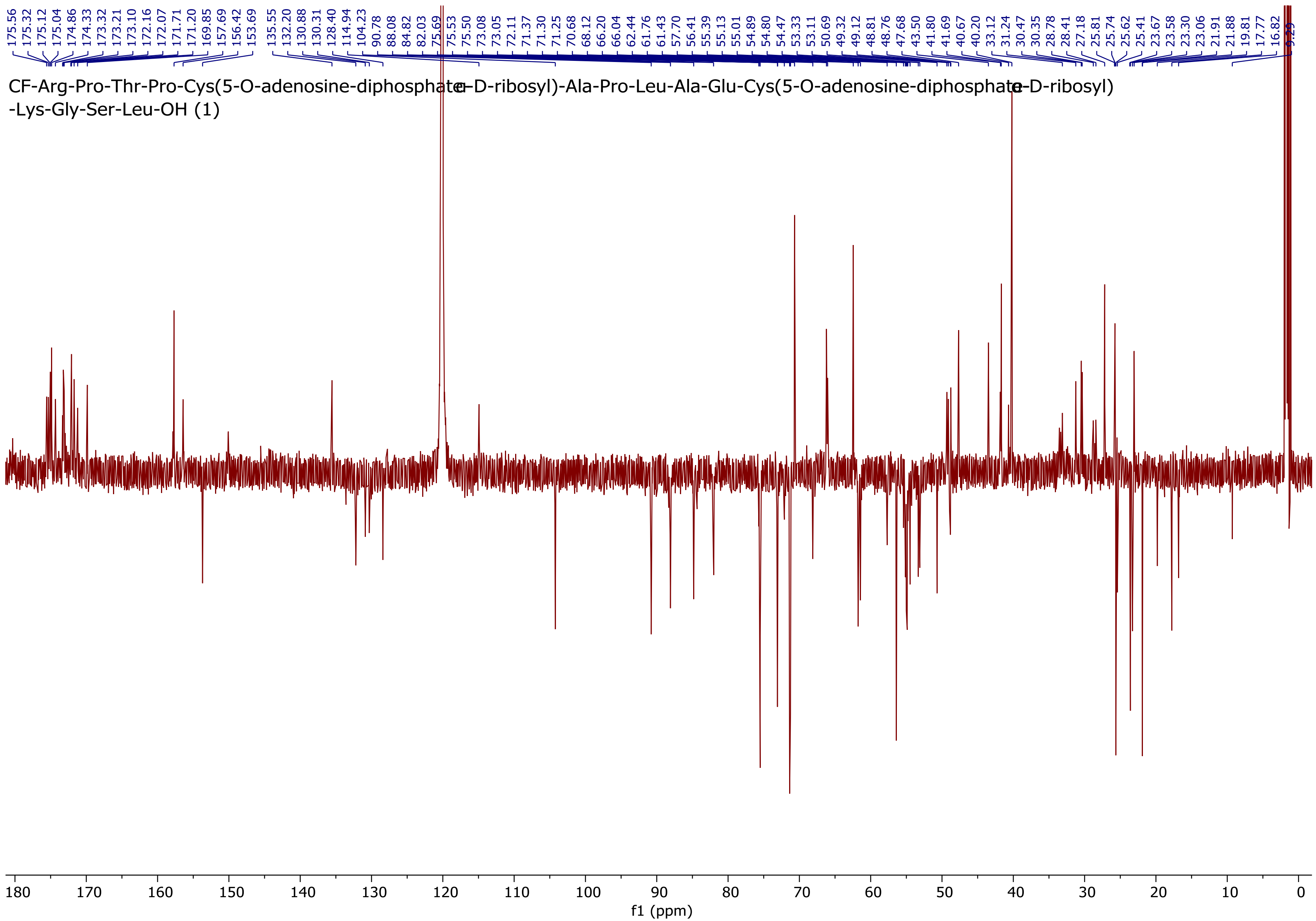

175.56
175.32
175.12
175.04
174.86
174.33
173.32
173.21
173.10
172.16
172.07
171.71
171.20
169.85
157.69
156.42
153.69
135.55
132.20
130.88
130.31
128.40
114.94
104.23
90.78
88.08
84.82
82.03
75.69
75.53
75.50
73.08
73.05
72.11
71.37
71.30
71.25
70.68
68.12
66.20
66.04
62.44
61.76
61.43
57.70
56.41
55.39
55.13
55.01
54.89
54.80
54.47
53.33
53.11
50.69
49.32
49.12
48.81
48.76
47.68
43.50
41.80
41.69
40.67
40.20
33.12
31.24
30.47
30.35
28.78
28.41
27.18
25.81
25.74
25.62
25.41
23.67
23.58
23.30
23.06
21.91
21.88
19.81
17.77
16.82
9.29
CF-Arg-Pro-Thr-Pro-Cys(5-O-adenosine-diphosphate-α-D-ribosyl)-Ala-Pro-Leu-Ala-Glu-Cys(5-O-adenosine-diphosphate-α-D-ribosyl)
-Lys-Gly-Ser-Leu-OH (1)
180
170
160
150
140
130
120
110
100
90
80
70
60
50
40
30
20
10
0
f1 (ppm)

CF-Arg-Pro-Thr-Pro-Cys(5-O-adenosine-diphosphate-α-D-ribosyl)-Ala-Pro-Leu-Ala-Glu-Cys(5-O-adenosine-diphosphate-α-D-ribosyl)
-Lys-Gly-Ser-Leu-OH (1)

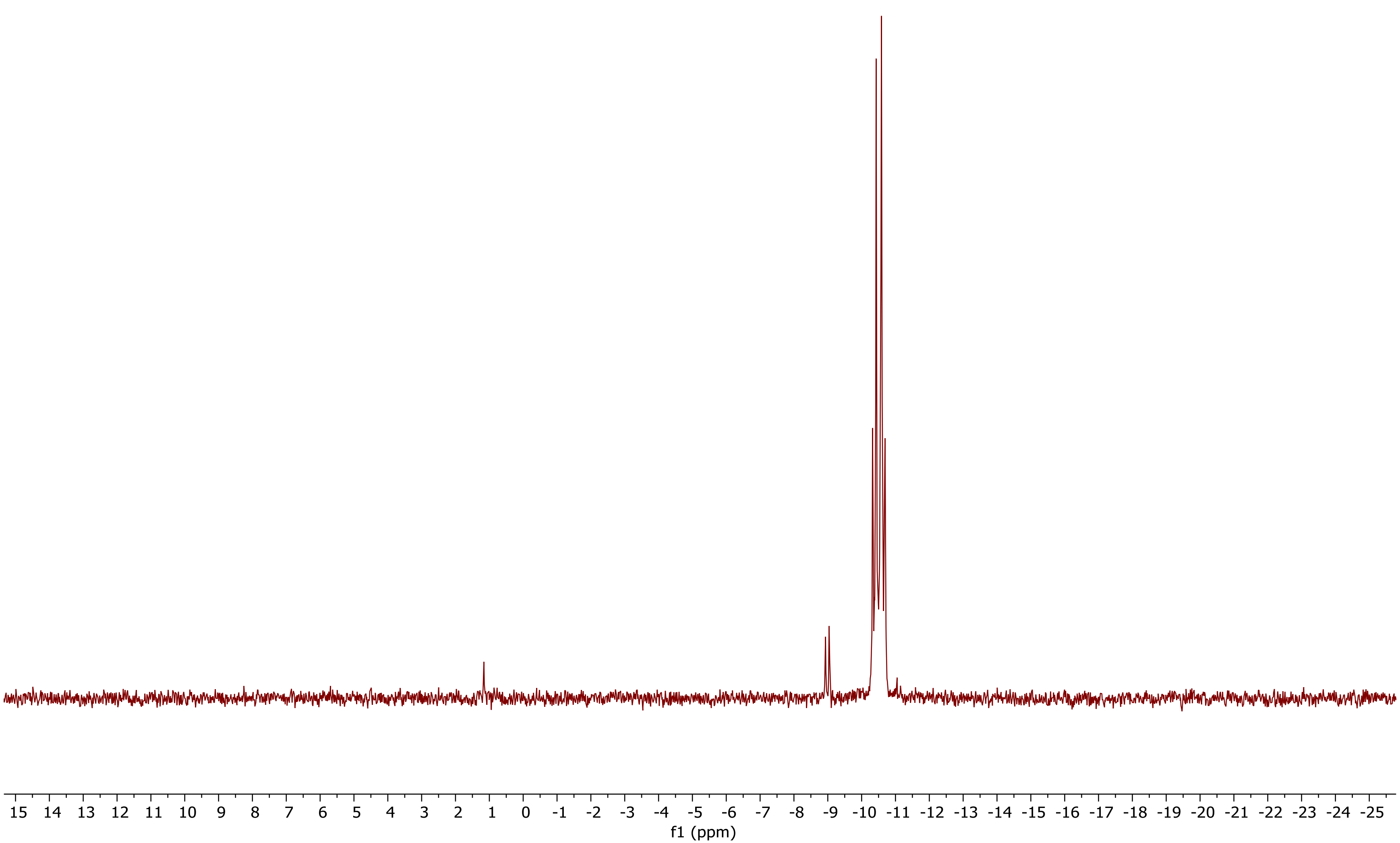

CF-Arg-Pro-Thr-Pro-Cys-Ala-Pro-Leu-Ala-Glu-Cys(5-O-adenosine-diphosphate-α-D-ribosyl)-Lys-Gly-Ser-Leu-OH (4)

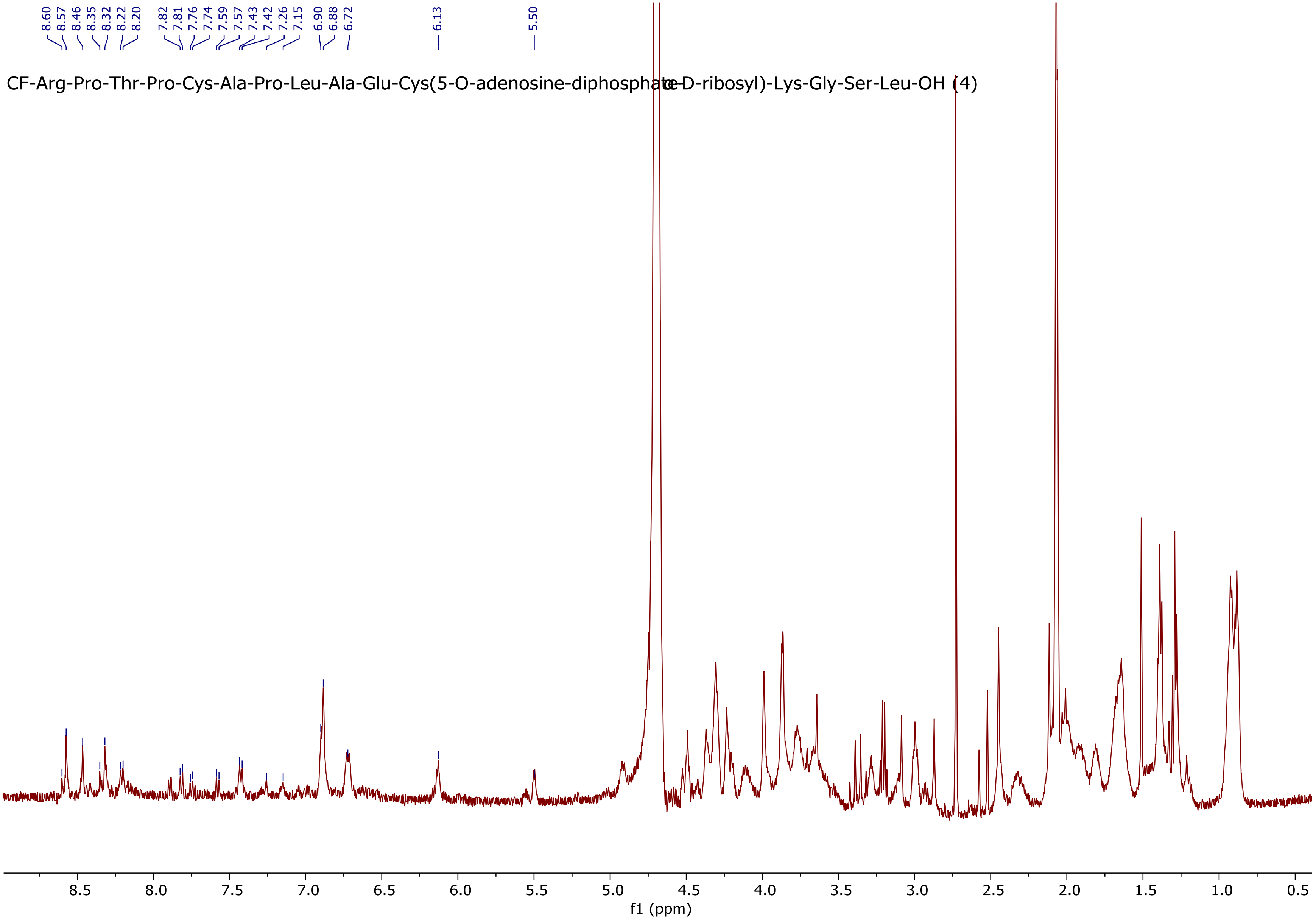

CF-Arg-Pro-Thr-Pro-Cys-Ala-Pro-Leu-Ala-Glu-Cys(5-O-adenosine-diphosphate-α-D-ribosyl)-Lys-Gly-Ser-Leu-OH (4)

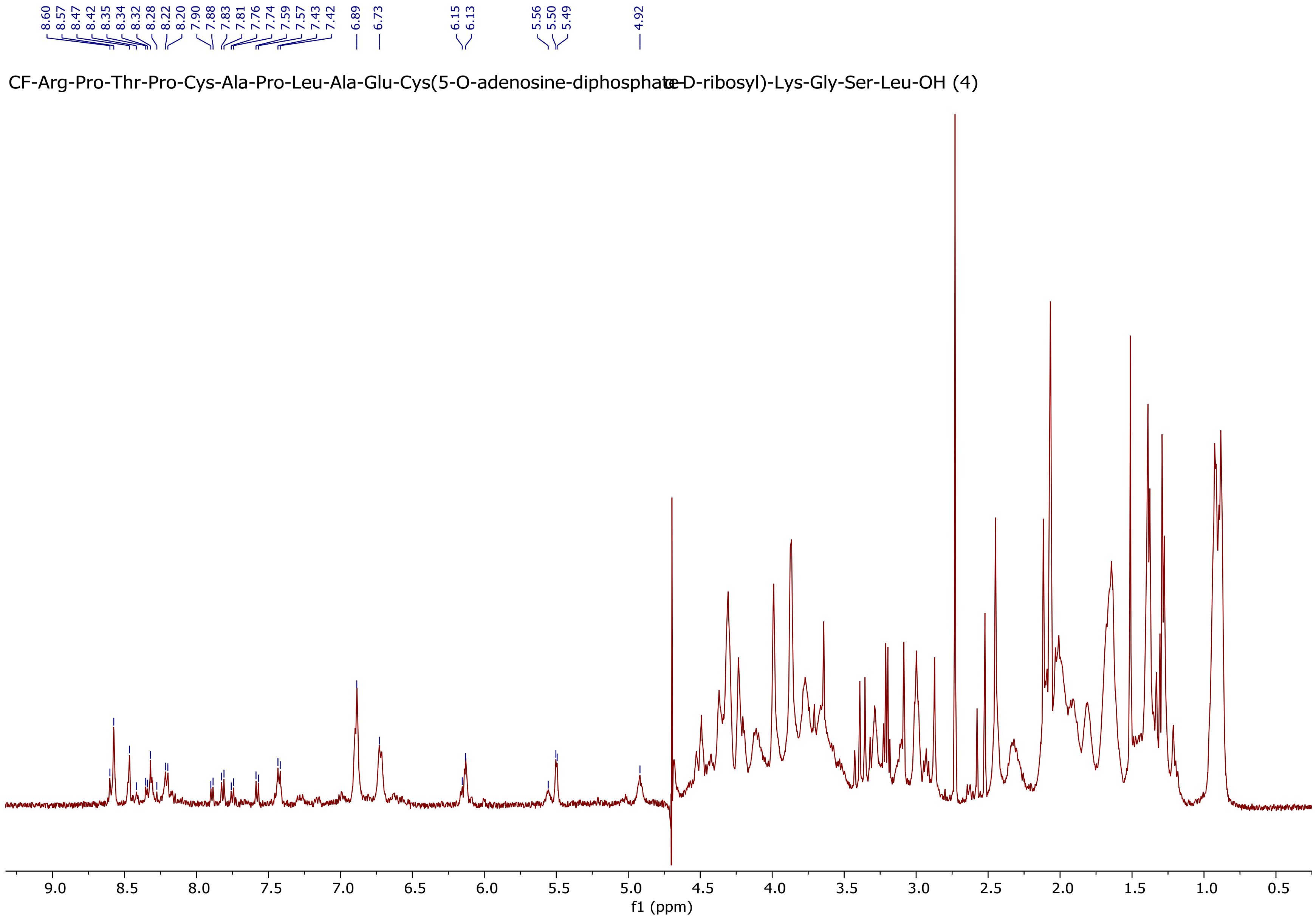

CF-Arg-Pro-Thr-Pro-Cys-Ala-Pro-Leu-Ala-Glu-Cys(5-O-adenosine-diphosphate-α-D-ribosyl)-Lys-Gly-Ser-Leu-OH (4)

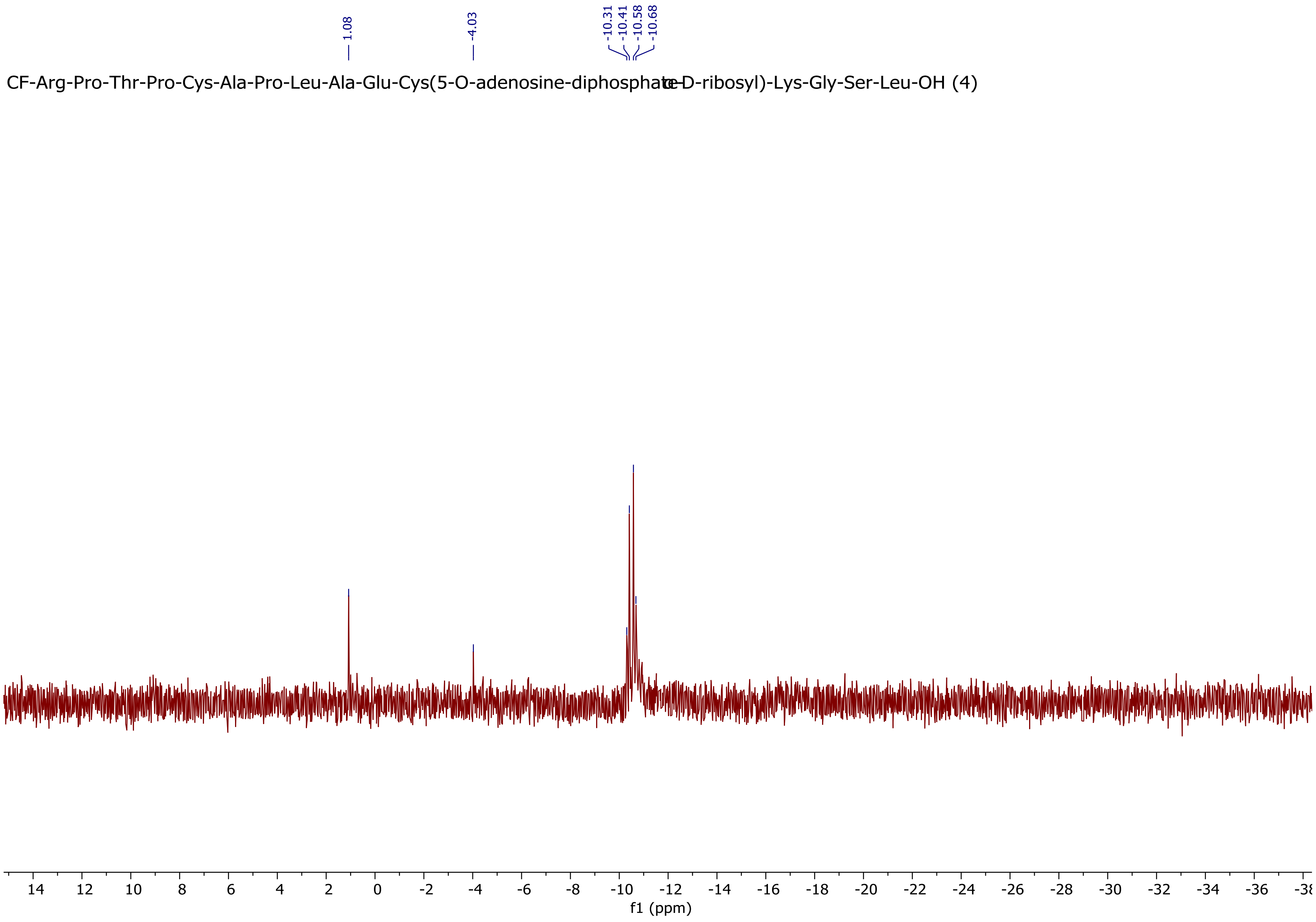

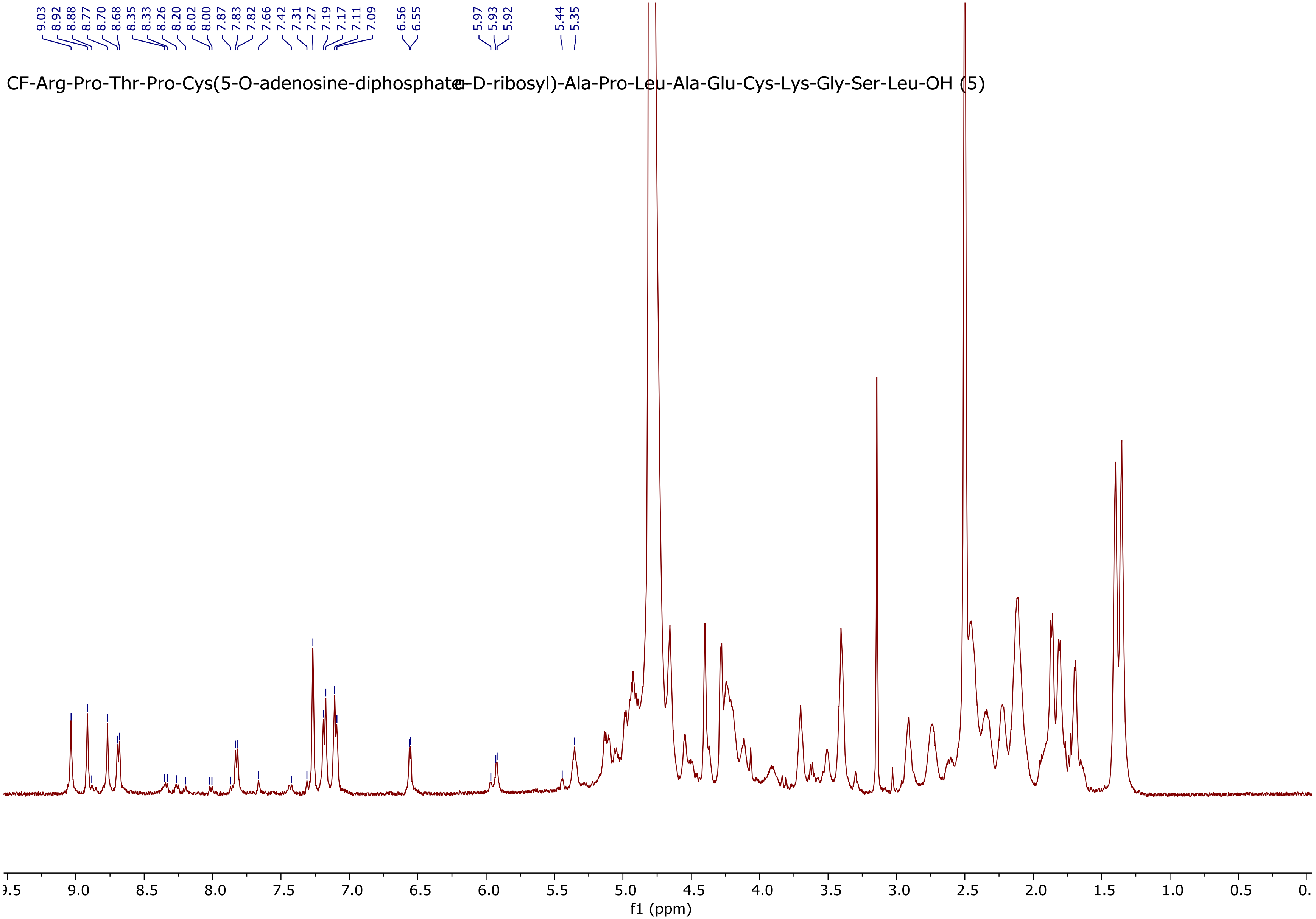
9.03 8.92 8.88 8.77 8.70 8.68 8.35 8.33 8.26 8.20 8.02 8.00 7.87 7.83 7.82 7.66 7.42 7.31 7.27 7.19 7.17 7.11 7.09 6.56 6.55 5.97 5.93 5.92 5.44 5.35
CF-Arg-Pro-Thr-Pro-Cys(5-O-adenosine-diphosphate-α-D-ribosyl)-Ala-Pro-Leu-Ala-Glu-Cys-Lys-Gly-Ser-Leu-OH (5)
9.0 8.5 8.0 7.5 7.0 6.5 6.0 5.5 5.0 4.5 4.0 3.5 3.0 2.5 2.0 1.5 1.0 0.5
f1 (ppm)

CF-Arg-Pro-Thr-Pro-Cys(5-O-adenosine-diphosphate-α-D-ribosyl)-Ala-Pro-Leu-Ala-Glu-Cys-Lys-Gly-Ser-Leu-OH (5)

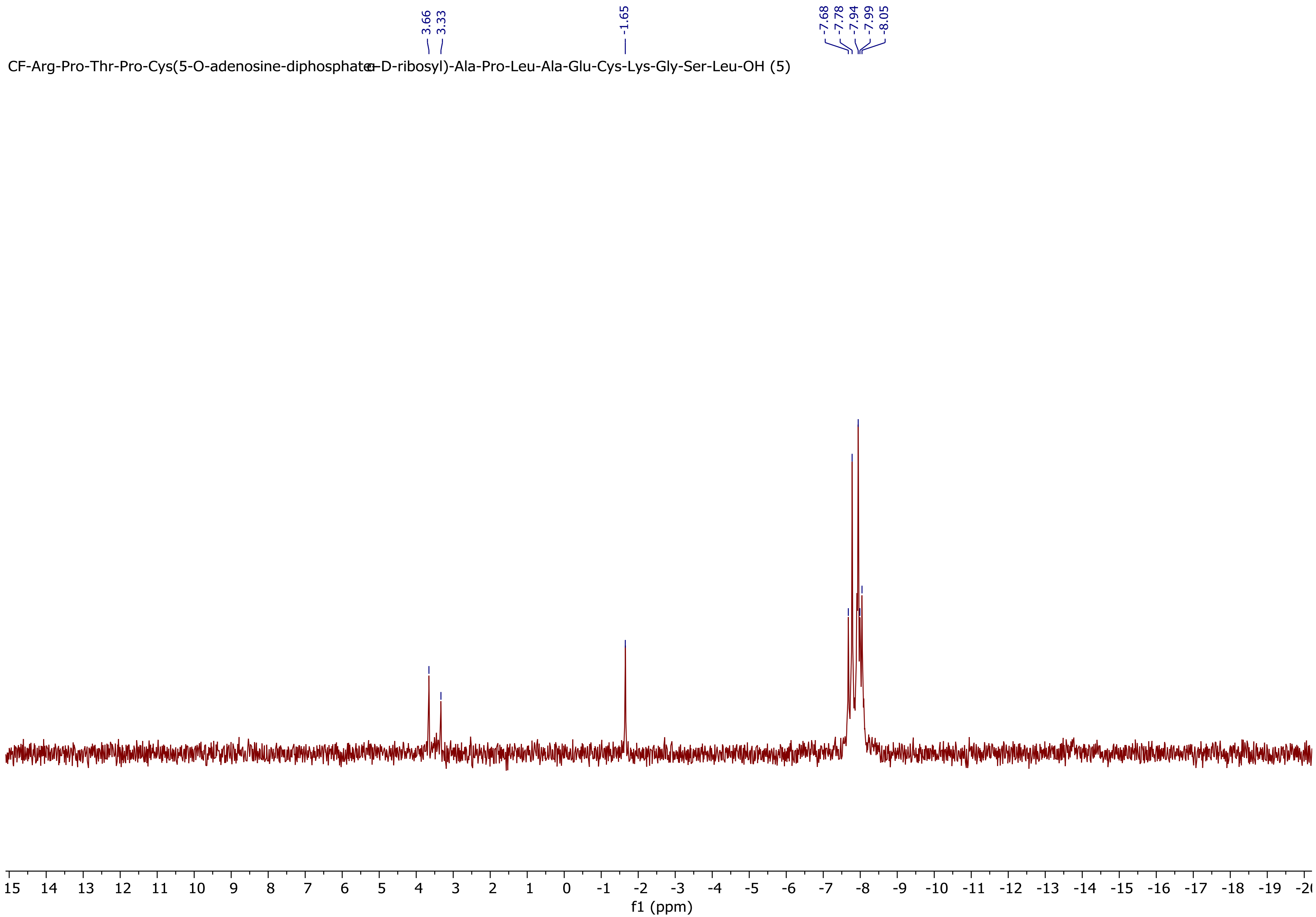

# References


(1) Voorneveld, J.; Rack, J. G. M.; van Gijlswijk, L.; Meeuwenoord, N. J.; Liu, Q.; Overkleeft, H. S.; van der Marel, G. A.; Ahel, I.; Filippov, D. V. Molecular Tools for the Study of ADP-Ribosylation: A Unified and Versatile Method to Synthesise Native Mono-ADP-Ribosylated Peptides. *Chem. - A Eur. J.* **2021**, *27*, 1–8. https://doi.org/10.1002/chem.202100337.

(2) Hakimelahi, G. H.; Proba, Z. A.; Ogilvie, K. K. New Catalysts and Procedures for the Dimethoxytritylation and Selective Silylation of Ribonucleosides. *Can. J. Chem.* **1982**, *60* (9), 1106–1113. https://doi.org/10.1139/v82-165.

(3) Hananya, N.; Daley, S. K.; Bagert, J. D.; Muir, T. W. Synthesis of ADP-Ribosylated Histones Reveals Site-Specific Impacts on Chromatin Structure and Function. *J. Am. Chem. Soc.* **2021**, *143* (29), 10847–10852. https://doi.org/10.1021/jacs.1c05429.
